# A rigorous and versatile statistical test for correlations between stationary time series

**DOI:** 10.1371/journal.pbio.3002758

**Published:** 2024-08-15

**Authors:** Alex E. Yuan, Wenying Shou

**Affiliations:** 1 Molecular and Cellular Biology PhD program, University of Washington, Seattle, Washington, United States of America; 2 Fred Hutchinson Cancer Center, Seattle, Washington, United States of America; 3 Centre for Life’s Origins and Evolution, Department of Genetics, Evolution and Environment, University College London, London, United Kingdom; Centre National de la Recherche Scientifique, FRANCE

## Abstract

In disciplines from biology to climate science, a routine task is to compute a correlation between a pair of time series and determine whether the correlation is statistically significant (i.e., unlikely under the null hypothesis that the time series are independent). This problem is challenging because time series typically exhibit autocorrelation and thus cannot be properly analyzed with the standard iid-oriented statistical tests. Although there are well-known parametric tests for time series, these are designed for linear correlation statistics and thus not suitable for the increasingly popular nonlinear correlation statistics. There are also nonparametric tests that can be used with any correlation statistic, but for these, the conditions that guarantee correct false positive rates are either restrictive or unclear. Here, we describe the truncated time-shift (TTS) test, a nonparametric procedure to test for dependence between 2 time series. We prove that this test correctly controls the false positive rate as long as one of the time series is stationary, a minimally restrictive requirement among current tests. The TTS test is versatile because it can be used with any correlation statistic. Using synthetic data, we demonstrate that this test performs correctly even while other tests suffer high false positive rates. In simulation examples, simple guidelines for parameter choices allow high statistical power to be achieved with sufficient data. We apply the test to datasets from climatology, animal behavior, and microbiome science, verifying previously discovered dependence relationships and detecting additional relationships.

## Abstract 2

Testing the significance of a correlation between time series without substantial assumptions is a longstanding challenge. This study presents the truncated time-shift (TTS) test, a conservative significance test that is valid as long as one time series is statistically stationary.

## Introduction

Researchers routinely look for correlations between variables to identify potentially important relationships or to use as a starting point for downstream modeling and experiments. In fields such as climatology, ecology, and physiology, data are often collected as time series, and so correlation analyses using time series are common.

Interpreting a correlation between time series can be challenging because it is easy to obtain a seemingly high correlation between 2 time series that have no meaningful relationship [1,2]. For example, consider 2 independent copies of an oscillatory system such as the abundance of prey in a classic Lotka–Volterra predator–prey model. One can show by simulation that independent replicates are often correlated across time, even without shared ecological drivers or fixed initial conditions (Fig A-1 in S1 Text). To avoid being fooled by spurious correlations, it helps to distinguish between the concepts of “correlation” and “dependence” and how each relates to causation. In time series research, “correlation” is often defined procedurally [1,3,4]; i.e., a correlation function is any function that takes a pair of time series and returns a number. We call this number a correlation statistic, and it is usually interpreted as a measure of similarity or relatedness. Examples include Pearson correlation coefficient, local similarity [5], and cross-map skill [6]. Whereas a correlation statistic is a description of an observed dataset, statistical dependence (or independence) is a hypothesis about the relationship between variables.

Two variables *x* and *y* are (statistically) dependent if the probability distribution of *x* while statistically controlling for *y* (the conditional distribution of *x* given *y*) differs from the distribution of *x* while not controlling for *y* (the marginal distribution of *x*). For example, lung cancer and smoking are dependent if the probability of lung cancer among smokers is different from that in the entire population. The concept of dependence applies not only to pairs of univariate variables but also to pairs of vectors such as time series. Importantly, dependence is linked to causality (as defined in the usual sense: *x* causes *y* if perturbations in *x* can alter *y*). The link between dependence and causality is due to Reichenbach’s common cause principle, which states that if 2 variables are dependent, then they are causally related: Either they share a common cause, or one variable causes the other (possibly indirectly) [7,8]. Thus, before seizing upon causal explanations, it is useful to first test whether an observed correlation is strong enough to indicate dependence.

To test against the null hypothesis of independence, 2 ingredients are needed: (i) a correlation statistic; and (ii) a method to estimate the distribution of that statistic under the null hypothesis of independence. This article focuses on the second ingredient.

In the simpler (nontemporal) case where measurements of 2 variables are independent and identically distributed (iid), the permutation test provides a general way to test for dependence between the 2 variables. Specifically, the permutation test randomly shuffles the index of one of the variables and then recomputes the correlation. This process is then repeated many (*N*_randomized_) times, essentially producing a null distribution. Under the null hypothesis that *x* and *y* are independent, correlations obtained from the original and the shuffled data follow the same distribution. Thus, a *p*-value can be calculated as (see, for example, section 6.2.5 of [9]):

$$p=\frac{N_{\geq }+1}{N_{randomized}+1}$$ (1)

where *N*_≥_ is the number of randomized correlations that are at least as strong as the original correlation. The “+1” terms account for the original correlation. This test has 3 especially desirable properties. First, the test is valid [9,10]: If dependence is inferred only when *p* is less than some significance level *α*, then the false positive rate (i.e., the chance of reporting dependence when it does not exist) will be no more than *α*. Second, the test is distribution-free: It does not require that the data or the correlation statistic follow a particular probability distribution [11]. Lastly, the test is without critical parameters: Its validity does not depend on any parameters that must be estimated or chosen by the user.

Dependence testing is less straightforward when applied to a pair of time series. Although a time series can have many data points, these data are not independent of each other in the sense that they are often autocorrelated (e.g., what occurs today influences what occurs tomorrow). A permutation test carried out by shuffling data within time series will generally not be valid. This is because temporal shuffling destroys autocorrelation, which often leads to artificially weak randomized correlations and thus an unacceptably high false positive rate (e.g., Fig 2 in [12]). If multiple independent and identical systems (trials) are available, we can instead perform a valid test by comparing within-trial correlations to between-trial correlations [12–14]. However, many important questions focus on a single pair of time series only (i.e., they have the “*n*-of-one” challenge). For example, global-scale environmental studies are necessarily *n*-of-one because replicate Earths do not exist, and the *n*-of-one perspective has been advocated in psychology because statistical patterns within one individual might differ from patterns in other individuals [15].

If a correct model of the autocorrelation happens to be known, this model can sometimes be used to remove the autocorrelation (“prewhitening”) so that standard correlation tests may be applied [16]. However, simulation benchmarks have shown that prewhitening-based dependence tests can have worryingly high false positive rates [17], and certain data types cannot be prewhitened [18].

An alternative approach to testing for dependence between time series is to use a test that takes autocorrelation into account (rather than eliminating it during data preprocessing). One can do so via either a parametric or nonparametric test. Parametric tests assume that the data follow a particular distribution (e.g., Gaussian) and use this assumption to analytically derive a null distribution for a particular statistic (such as Pearson correlation coefficient) [17,19–21]. However, an analytical null distribution is not available for some increasingly popular statistics such as cross-map skill [6,22–26] (see also [13,27] for a broader overview of nonlinear dependence statistics).

When parametric tests are unavailable or inappropriate, it is common to test for dependence between time series by using a nonparametric approach called surrogate data testing [27]. Since they do not rely on an analytically derived null distribution, surrogate data tests can accommodate any correlation statistic. This approach begins with 2 time series {*x*_1_,*x*_2_,…,*x*_*n*_} and {*y*_1_,*y*_2_,…,*y*_*n*_} (abbreviated {*x*_*t*_} and {*y*_*t*_}) and computes some measure of correlation between them. Next, one uses a computer to simulate independent replicates of {*y*_*t*_}. These simulated {*y*_*t*_} time series are called “surrogate” {*y*_*t*_} series. Finally, one computes the correlation between {*x*_*t*_} and each surrogate {*y*_*t*_}. A *p*-value is then given by the proportion of surrogate {*y*_*t*_} series that produce a correlation equal to or larger than the real {*y*_*t*_}. More precisely, the *p*-value is given by Eq 1, but where *N*_≥_ is now the number of surrogates that produce a correlation statistic at least as large as the original {*y*_*t*_} series and *N*_*randomized*_ is the total number of surrogates [28].

Several procedures have been used to generate time series surrogates, each with different strengths and limitations. For example, the random phase procedure decomposes the {*y*_*t*_} time series into sine waves, randomly shifts these sine waves in time, and finally takes their sum to produce surrogates [23,29]. This procedure is valid when {*y*_*t*_} is a Gaussian process (meaning that any subsequence follows a multivariate Gaussian distribution) and is stationary (meaning that this distribution does not change over time [13,27]; see [30] for precise validity conditions). A more general version of the random phase procedure, called the iterative amplitude-adjusted Fourier transform (IAAFT) procedure, is valid when {*y*_*t*_} is a stationary Gaussian process that has been passed through an invertible but possibly nonlinear “measurement function” [27,28]. Yet, even this more general condition would seem to exclude processes that were never Gaussian to begin with. Surrogates can also be produced by a block bootstrap method in which random subsequences are selected from {*y*_*t*_} and joined together [31,32]. However, junctions between the blocks can produce disruptions, rendering the test inexact [31]. A sophisticated variant of the block bootstrap method, called the twin method, attempts to position blocks so that the disruptions are minimized [33]. However, even for this method, validity depends on “embedding parameters” [34] that must be appropriately chosen by the user, a potentially difficult task [35]. Overall, such surrogate procedures do not embody the 3 desirable properties listed above (being valid, distribution-free, and without critical parameters).

The procedure we propose in this article is most similar to a class of “time-shift” procedures, which produce surrogates by shifting the original {*y*_*t*_} in time [13,24,32,36–39]. One can in principle use these surrogates to calculate a *p*-value according to Eq 1. However, Bartlett [40] noted that such an approach is generally invalid because the surrogates are statistically dependent on each other.

Here, we describe a test for dependence between 2 time series that we call the truncated time-shift (TTS) test. We mathematically prove that the TTS test is valid as long as one of the 2 time series is strict-sense stationary. The TTS test is compatible with any correlation statistic, and its validity does not require the assumption of a particular probability distribution, nor does it require that a user correctly select some parameter. Although the statistical power of the TTS test can depend on user-selected parameters, we demonstrate that in common benchmark systems, simple guidelines for parameter choices allow high power to be achieved (as long as sufficient data are available). Lastly, we demonstrate the use of the TTS test by applying it to real data from climatology, animal behavior, and microbiome science.

We note that after we uploaded an earlier version of this manuscript to bioRxiv [41], we happened to discover an arXiv preprint that independently conceived and proved an equivalent test [18]. The preprint, whose primary focus is a description and proof of the TTS test, additionally shows that the TTS test is not excessively conservative; i.e., using the same procedure with a less stringent cutoff will produce a test that is potentially invalid for finite data (although a less stringent cutoff can sometimes be made valid in the limit of infinite data). We nevertheless provide our version of the proof in section 2 of S1 Text because (1) our proof is more complete in the sense that each statement is justified; (2) our proof applies more directly to finite-time processes, as we use a definition of stationarity that applies explicitly to finite time series (instead of stationarity in standard stochastic process literature, which is defined only for infinite time series [42–44]); and (3) our proof is intended to be relatively accessible, with graphical illustrations of intermediate lemmas and relevant background concepts (section 2.2 of S1 Text).

## Results

### The truncated time shift (TTS) test

We say that the temporal processes {*x*_*t*_} and {*y*_*t*_} are independent if

$$P(x_{1}\leq a_{1},\ldots ,x_{n}\leq a_{n})P(y_{1}\leq b_{1},\ldots ,y_{n}\leq b_{n})=P(x_{1}\leq a_{1},\ldots ,x_{n}\leq a_{n},y_{1}\leq b_{1},\ldots ,y_{n}\leq b_{n})$$

for all {*a*_*t*_} and {*b*_*t*_}. This is one of several equivalent definitions (see, e.g., pg. 127 in [45]).

The TTS test is designed to test for dependence between {*x*_*t*_} and {*y*_*t*_}, with the null hypothesis being that they are independent. The test is based on time-shifted surrogates and requires shifting the original {*y*_*t*_} series in time without altering its length. One way to achieve this is to use cyclic permutations [24,32]; i.e., if the original {*y*_*t*_} series were {1,2,3,4}, then there would be 3 surrogates, given by {2,3,4,1}, {3,4,1,2}, and {4,1,2,3}. However, these surrogates artificially force the first and final points of the original {*y*_*t*_} series to become neighbors, which can distort the dynamics [27].

Instead, we will truncate time series and then shift them to generate surrogates [27]. Starting with {*x*_1_,*x*_2_,…,*x*_*n*_}, we delete *r* time points from each end of the sequence and obtain

$$x^{trunc}=\{x_{1+r},x_{2+r},\ldots ,x_{n-r}\}.$$

**Fig 1:**
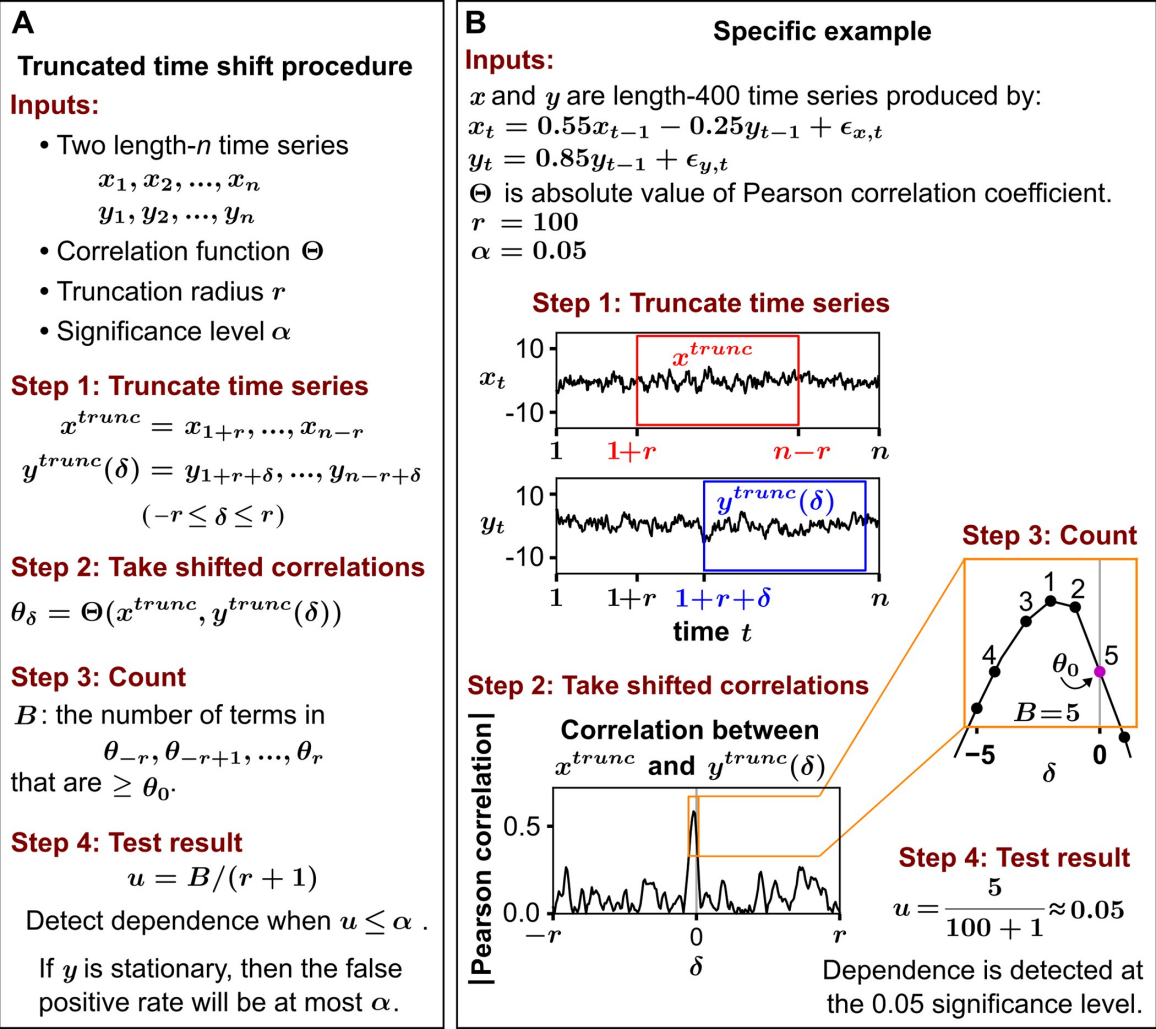
The truncated time shift procedure. (**A**) Stepwise description of the procedure. (**B**) A worked example. In the example, the process noise terms *ε*_*x*,*t*_ and *ε*_*y*,*t*_ are independent and identically distributed normal random variables with variance of 1 and 0 mean. Note that in step 4, the statistic *u* can exceed 1 since *B* has a maximum of 2*r*+1. If one insists on reporting a *u* value between 0 and 1, then min(*u*,1) instead of *u* can be used, giving what is sometimes called a “superuniform” *p*-value [46,47]. Technically, this procedure tests for dependence between {*y*_*t*_} and the truncated *x* window; if {*y*_*t*_} somehow depended only on *x*_*t*_ values outside the window (i.e., {*x*_1_, …, *x*_*r*_} and {*x*_*n*−*r*+1_, …, *x*_*n*_}), the dependence would likely go undetected since these *x*_*t*_ values are not used to calculate *u*.

We call *r* the “truncation radius.” We then define a collection of truncated and shifted *y* time series, which all have the same length as *x*^*trunc*^:

$$y^{trunc}(\delta )=\{y_{1+r+\delta },y_{2+r+\delta },\ldots ,y_{n-r+\delta }\}$$

where the shift *δ* can take on integer values between −*r* and *r* (Fig 1A and 1B, step 1). Note that *x*^*trunc*^ and *y*^*trunc*^(*δ*) are aligned when *δ* = 0. Thus, we can think of *y*^*trunc*^(0) as the original time series and *y*^*trunc*^(*δ*) for *δ* ≠ 0 as the surrogate time series. For each value of *δ* between −*r* and *r*, we compute the correlation between *x*^*trunc*^ and *y*^*trunc*^(*δ*) (Fig 1A and 1B, step 2). We then define *B* (for “Bigger”) as the number of shifts *δ* that produce a correlation at least as large as when *δ* = 0 (Fig 1A and 1B, step 3). *B* is bounded between 1 and 2*r*+1: We have *B* = 1 if the strictly greatest correlation is obtained when *δ* = 0. Conversely, *B* is equal to 2*r*+1 if the lowest correlation (or a correlation that is tied for lowest) is obtained when *δ* = 0.

If one were to naively apply the traditional logic of surrogate data testing (Eq 1), a *p*-value could be written as simply the proportion of correlations (shifted or not) that are at least as large as as the unshifted correlation:

$$p_{naive}=B/(2r+1).$$ (2)

As Bartlett [40] noted, *p*_*naive*_ is not a valid *p*-value because the surrogate *y* series are not independent of each other (e.g., 2 consecutive shifts are nearly identical). Instead, our approach relies upon the following statistic:

u=B/(r+1). (3)

We refer to this procedure as the TTS test. Although *u* is not a *p*-value in the usual sense (e.g., *u*>1 is possible), *u* can be used in the same way to establish evidence against the null hypothesis that {*x*_*t*_} and {*y*_*t*_} are independent; i.e., if the null hypothesis is true, then *P*(*u*≤*α*) is no more than *α* (Fig 1A and 1B, step 4). In section 2 of S1 Text, we prove that this property holds under the assumption that *y* is stationary. Roughly speaking, a temporal process is stationary (also called strict-sense stationary) if its probability distribution does not change over time (see section 2.2 of S1 Text for a precise mathematical definition). Stationary processes include many equilibrium processes, noise processes, chaotic processes, and periodic processes with random phases.

The above mathematical result may also provide a touch of comfort to analyses performed using the naive test: Comparing the formulas for *u* and *p*_*naive*_ (Eq 3 and Eq 2), we can see that as long as the requirement of the TTS test is satisfied (i.e., the time series used to generate surrogate data is stationary), the false positive rate of the naive test will not be inflated above the significance level by more than a factor of 2. However, many applied studies do not use the naive TTS test as we have described it but instead use a number of variations on it [32,36,38,39]. In section 3 of S1 Text, we consider 2 possible variants of the naive TTS test and use simulation examples to show that these can in principle be miscalibrated by far more than 2-fold.

### The TTS test correctly controls false positive rates when similar tests do not

Here, using simulated systems consisting of pairs of independent time series, we compare the false positive rates for the TTS test and several existing surrogate data tests, as well as 2 versions of a parametric test. Whereas all other tests fail in at least 1 stationary system, the TTS test performs correctly in all stationary systems, as expected. The TTS test also performs well in the 2 nonstationary systems considered here.

**Fig 2:**
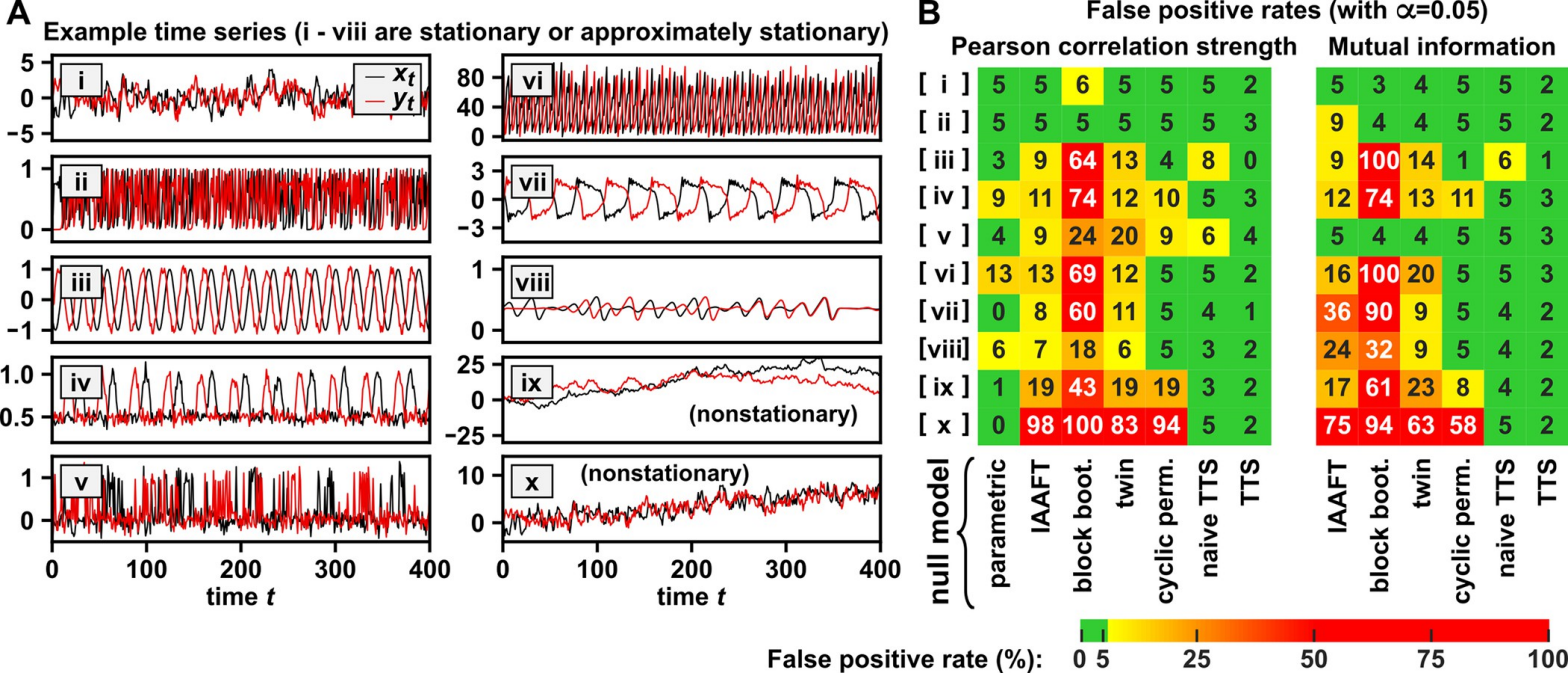
The TTS test controls the false positive rate in stationary systems and some nonstationary systems. For each system, 2 independent time series were simulated and correlated using either the absolute value of the Pearson correlation coefficient or an estimator of mutual information. (**A**) Example time series from different benchmark systems, some of which (i.e., systems i–vi) are provably stationary (section 4 of S1 Text). Systems vii–viii are probably approximately stationary, but stationarity proofs are difficult for multivariate nonlinear stochastic systems [50]. Systems ix–x are nonstationary. A system can have process noise (whose effects propagate to subsequent time steps) and/or measurement noise (whose effects do not propagate). (i) First-order autoregressive process: Current values depend linearly on values one step ago and process noise. (ii) Logistic map: A deterministic discrete-time model of population dynamics with growth and a carrying capacity [51]. (iii) Two sine waves with independent random phases. The *x* time series is noiseless, while the *y* time series has measurement noise whose strength varies with a low-frequency phase-randomized “sawtooth wave” [52]. (iv) Sine waves with independent random phases, with a detection threshold and measurement noise. (v) A series of coin flips with measurement noise (with “heads” and “tails” coded as 1 and 0, respectively), where coin flips are autocorrelated because the probability of a “heads” varies over time in a stationary way. (vi) A model of a population with exponential growth, periodic extinction events, and constant immigration, with a random wait time between initialization and data collection. (vii) A stochastic discrete-time FitzHugh–Nagumo model, which is a nonlinear oscillator inspired by neural voltage dynamics. (viii) Chaotic Lotka–Volterra model: an ecological model that includes intra- and interspecies competition. Data were collected after waiting 10^4^ or 5×10^3^ time steps for system vii or viii, respectively, to allow each system to relax to a stationary equilibrium. (ix) A random walk with Gaussian steps (i.e., *x*_*t*_−*x*_*t*−1_ has a normal distribution with a mean of 0). (x) The same process as in (i), but with an additive temporal trend. (**B**) False positive rates are calculated from 1.5×10^4^ trials. Each test was then used to assess significance (at the 0.05 level) of the correlation under the null hypothesis of independence. The labels “block boot.” and “cyclic perm.” are shorthand for stationary block bootstrap and cyclic permutation surrogates. See section 4 of S1 Text for further details. The numerical values shown in this figure and the code used to produce the figure are in S1 Data and S1 Code, respectively.

Systems are shown in Fig 2A and indexed by Roman numerals. Systems i and ii are a first-order autoregressive process and the logistic map, which are 2 stationary systems commonly used for benchmarking (e.g., [6,29]). Systems iii to vi are 4 stationary systems with a combination of periodic dynamics and noise designed to challenge existing tests. See section 4.1 of S1 Text for brief proofs of stationary for systems i to vi. Systems vii and viii are 2 biologically inspired nonlinear systems: a stochastic FitzHugh–Nagumo neuronal model [48] and a competitive Lotka–Volterra system with chaotic behavior [49]. We expect that systems vii and viii are also stationary, although proofs are difficult for multivariate nonlinear systems [50]. Instead, we provide numerical evidence in section 4.2 of S1 Text that suggests systems vii and viii are stationary and that verifies the stationarity of systems i to vi. Systems ix and x are both nonstationary: a random walk and a first-order autoregressive process (same as system i) with an additive term that increases over time. In all cases, the 2 time series are independent by construction (mathematical details in section 4 of S1 Text).

Two different correlation statistics were used: (i) Pearson correlation strength (the absolute value of the sample Pearson correlation coefficient; Fig 2B, left half), which is a linear form of correlation; and (ii) an estimator of mutual information [53], which is a popular nonlinear form of correlation (Fig 2B, right half). We do not use statistics based on the Granger causality framework as correlation statistics in this paper. This is because the Granger causality framework requires tests of conditional dependence [54], whereas the TTS test and most surrogate data procedures provide tests of (unconditional) dependence and thus are generally inappropriate for Granger causality testing (i.e., Fig 6 of [55]).

For each statistic, we compared the following surrogate data tests: IAAFT [28], stationary block bootstrap [31,32,56], twin [33], cyclic permutation, naive TTS test (Eq 2), and TTS test (Fig 1). For 3 of these tests (IAAFT, stationary block bootstrap, and cyclic permutation), we used circularization to reduce a potential discontinuity caused by wrap-around effects (Methods), as recommended by [13,28]. We also assessed 2 parametric testing procedures: a *t* test for Pearson correlation strength given by [19] and a modified version of the same test suggested by [20] (Methods). In this benchmark, the latter test exceeded the 5% false positive rate threshold more often than the first test. For this reason, we show only the original test of [19] in Fig 2 and use only this test as our parametric test for all subsequent comparative analyses.

For all stationary systems, the TTS test has a false positive rate that does not exceed 5%, as expected given our proof in section 2 of S1 Text. All procedures other than the TTS test mistakenly detect dependence at rates above 5% in one or more stationary systems (Fig 2B). In section 4.5 of S1 Text, we examine possible reasons behind failure modes of the cyclic permutation procedure, which is perhaps the the closest relative of the TTS test in common use [24,32,38]. The TTS test also showed low false positive rates with the 2 nonstationary systems (Fig 2B, last 2 rows), although it is not guaranteed to be valid when surrogates are generated from a nonstationary process (e.g., Fig A-13 in S1 Text).

For time series that can be decomposed into a deterministic “trend” and a stationary component (e.g., system x of Fig 2), the TTS test can be modified to be rigorous by first detrending followed by retrending (similar to [57]). The basic idea is to (1) extract the stationary component by removing the trend; (2) generate surrogates from the stationary component; and then (3) add the trend back to the surrogates. In section 5 of S1 Text, we demonstrate that this detrending–retrending TTS procedure produces a valid surrogate data test for time series that are nonstationary due to a deterministic trend.

To summarize, using simulation examples, we have shown that the TTS test is universally valid in stationary time series and that the same cannot be said of several popular surrogate data tests.

### Strategies to achieve high statistical power with the TTS test

Here, we consider the TTS test’s power (i.e., true positive rate). In many settings, we may intuitively expect that 2 dependent time series should have a high correlation at 0 shift or small shifts and that this correlation should decay to 0 at large shifts. Thus, if an appropriate correlation statistic is used and if sufficient data are available, the TTS test should reliably detect dependence. Indeed, we explore this idea via an informal mathematical argument in section 6 of S1 Text. Below, we discuss 2 factors that impact the power of the TTS test and then demonstrate with simulations.

The power of the TTS test is affected by the truncation radius *r*, which simultaneously determines the length of the truncated time series (which is *2r* less than the total number of time points) and the number of time-shifted surrogates (which is *2r*). More precisely, a rearrangement of Eq 3 gives *r* = *B*/*u*−1. If *r* is exactly 19, significance will be detected at the *α* = 0.05 level only when *B* = 1, meaning that the unshifted correlation would need to exceed all shifted correlations. If 20≤*r*≤38, significance at the 0.05 level still requires *B* = 1 (since if *B* = 2 and *r* = 38, then *u* = *B*/(*r*+1) = 2/39>0.05). Thus, choosing 20≤*r*≤38 is unlikely to achieve higher power than setting *r* = 19. Generalizing this reasoning, for a significance level *α*, power is maximized when

r=j/α−1 (4)

where *j* is a positive integer (Fig A-14 in S1 Text). However, Eq 4 does not specify *j*. In general, a larger *r* provides more null (i.e., time-shifted) correlations to which we may compare the unshifted correlation, which generally increases power [58]. However, a larger *r* also means fewer data for calculating the correlation statistic, which could make the statistic noisier, reducing power.

**Fig 3:**
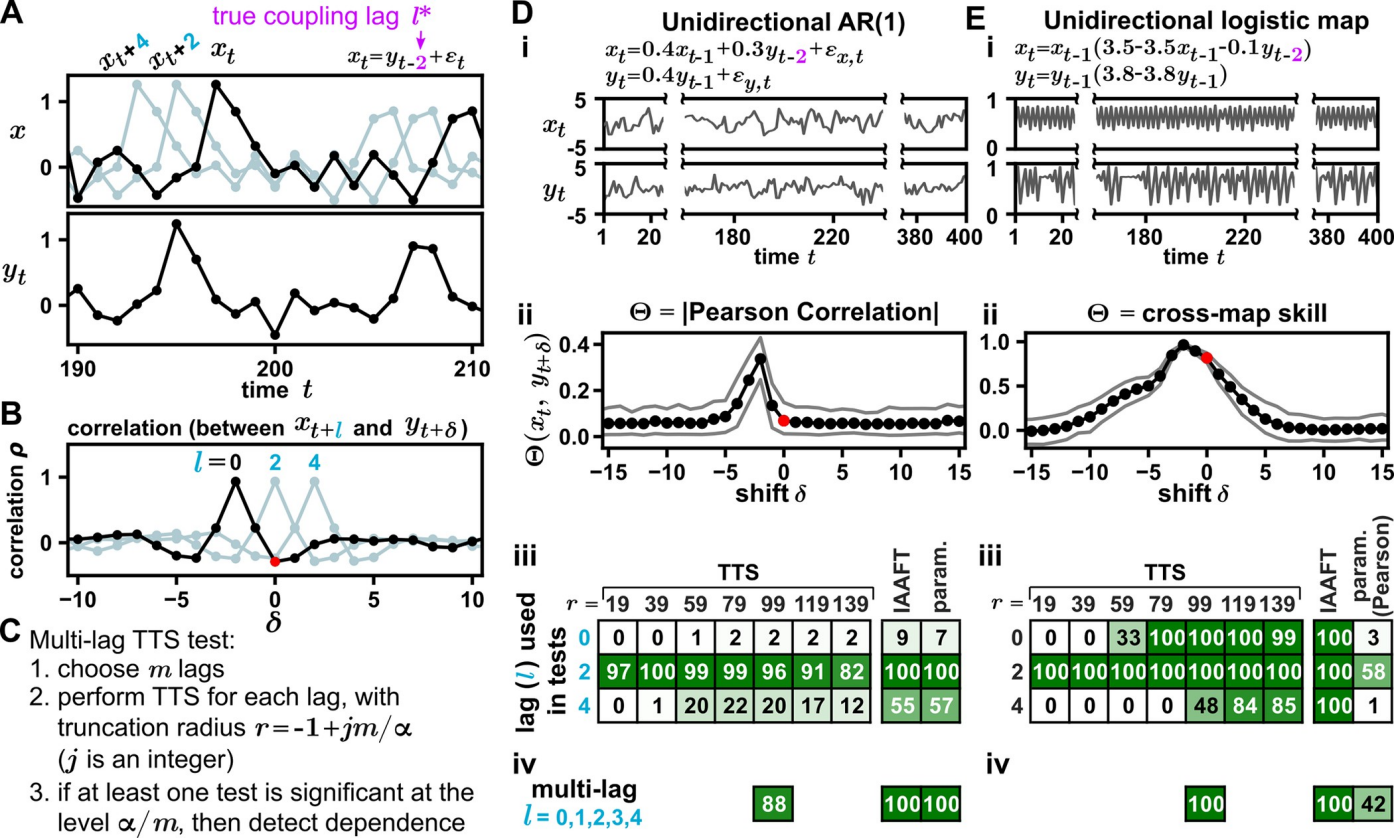
Strategies for increasing power in the presence of a coupling lag. (**A-C**) Conceptual illustration of incorporating lags into TTS tests. (**D, E**) Various strategies to account for a coupling lag can increase the power of the TTS test. (**i**) Time series in which *y* influences *x* with a lag of 2. We compare a coupled linear autoregressive process (D) and a nonlinear logistic map—a model inspired by ecological competition [6,59] (E). (**ii**) Shifted correlation plots when no lag is incorporated into the test. The absolute value of the Pearson correlation (D) or cross-map skill (E) was computed between *x*_*t*_ and *y*_*t*+*δ*_ for various shifts *δ*. Detecting dependence in D is more challenging than in E since the unshifted correlation (red dot) is at the foothill in D but near the maximum in E. To calculate cross-map skill, we used *x* to estimate *y* (as appropriate when *y* influences *x* [6]), and the embedding dimension and the embedding lag were set to 2 and 1, respectively, following prior works [6,59]. Black dots indicate the mean correlation (and grey lines indicate the upper and lower 10%) from 100 trials with a truncation radius of 79. (**iii**, **iv**) Power comparison. Tests in D-iii and E-iii used the correlation statistic specified in D-ii and E-ii, respectively, except for the parametric test, which always used Pearson correlation. In the lag-*l* tests, we tested for dependence between {*x*_{1+*l*}_, …, *x*_{*n*}_} and {*y*_1_,…,*y*_*n*−*l*_}, where *l* = 0,2, or 4 for a single lag at significance level 0.05 (iii) or *l* = 0,1,2,3,4 for a multi-lag test at a Bonferroni-corrected significance level 0.05/5 = 0.01 (iv). Power was computed as the proportion of 10^4^ simulations in which dependence was detected. The numerical values shown in this figure and the code used to produce the figure are in S1 Data and S1 Code, respectively. AR(1), autoregressive process of order 1.

When there is lagged dependence between 2 variables (e.g., when one variable affects another variable after a lag or when a common driver affects 2 variables asynchronously), the power of TTS test can be compromised. This is because with lagged dependence, correlation is maximized at a shift away from 0, which can lead to a false negative. To avoid this, we may “preshift” 1 time series by *l* time units (Fig 3B) before applying the TTS test. More precisely, we say that the “lag-*l* TTS test” is the TTS test when applied to {*x*_1+*l*_,…,*x*_*n*_} and {*y*_1_,…,*y*_*n*−*l*_} if *l*≥0, or to {*x*_1_,…,*x*_*n*−*l*_} and {*y*_1+*l*_,…,*y*_*n*_} if *l*<0. We stress that *l* should not be selected based on the data themselves, since this way, dependence will always be declared. Rather, lags could ideally be determined based on a mechanistic hypothesis or prior exploratory pilot studies from a similar but independent system.

Fig 3D and 3E show how the power of the TTS test varies with truncation radius *r* and lag *l* in 2 systems where *y* influences *x* after a lag of 2: A linear autoregressive process (where the correlation statistic was the Pearson correlation strength) and a coupled logistic map (where the statistic was cross-map skill).

The choice of the lag *l* is often consequential. The optimal lag is the lag such that the correlation is more likely to be maximized at a shift of *δ* = 0 than any other shift. In Fig 3, the optimal lag is 2. If the optimal lag is chosen, then power is generally higher (unsurprisingly), and a small *r* (less than 20% of the time series length) appears to work well. For example, in the linear autoregressive process (Fig 3D), power is high when *r*<80 = 20%×400. In S3 Data, we vary the time series length in a similar system and show that *r* = 19 gives higher power than *r* = 79 whenever the length is ≤325. Similar results were obtained with a nonlinearly coupled autoregressive system (S5 Data).

If *l* is not chosen optimally, then by definition, the correlation is likely not maximized at *δ* = 0 so that the smallest previously usable *r* value of 1/*α*−1 will no longer enable us to detect dependence (per Eq 3, since *B*>1). In Fig 3, the shifted correlations resemble a hill with a peak at *δ* = −2. The correlations decay with distance from the peak until they are indistinguishable from baseline correlations even further away. In Fig 3D (subpanel iii, first row; *l* = 0), the unshifted correlation is not on the hill (i.e., it is not appreciably greater than other baseline correlations), and the power of the TTS test is low at all values of *r*. Alternatively, in Fig 3E (subpanel iii, first row; *l* = 0), the unshifted correlation is on the hill. In this case, the TTS test detects dependence when *r* is large enough (≥79 in this case) to account for the points on the hill above the unshifted correlation. In S7 Data (rows 8 to 12), we examine a similar system with variable coupling lags and observe that as the coupling lag increases, the optimal value of *r* also increases.

If we do not know the optimal value of *l* exactly but can pin it down to a range, we can perform a “multi-lag” test (Fig 3C): test for dependence between {*x*_*t*+1_} and {*y*_*t*_} for several different values of *l* and perform a Bonferroni correction. More precisely, if *m* different lags are tested, then dependence may be reported if any of the *m* tests is significant at the *α*/*m* level. Parallel to Eq 4, for *m* tests and a significance level of *α*, the optimal truncation radius *r* values are

r=jm/α−1 (5)

where *j* is a positive integer (Fig 3C). With a larger range of possible lags, the minimum *r* value increases and longer series may be needed. Note that because of the Bonferroni correction, the multi-lag procedure does not compromise the validity of the TTS test (S8 Data).

Subpanel iv of Fig 3D illustrates a scenario where the optimal *l* is actually 2 but is only known to be between 0 and 4. Whereas fortuitously choosing *l* = 2 leads to power above 80% for all *r* values tested, erroneously choosing *l* = 0 or 4 leads to unacceptably low power. Using the multi-shift test (Fig 3D, subpanel iv) and selecting *r* = 99 as per Eq 5 gives power of 88% without needing to correctly guess *l* = 2. S6 and S7 Data contain results from systems similar to those of Fig 3D and 3E but with longer time series and bidirectional coupling with randomly assigned interaction lags. In these settings, multi-lag TTS tests (with *r* set to the minimum value satisfying Eq 5) had high power, as long as the set of *l* values used covered the range of possible interaction lags.

How does the power of the TTS test compare to other tests? For comparison, Fig 3 includes the commonly used IAAFT test with the same correlation statistics as the TTS test, as well as the parametric test used in Fig 2 with the required Pearson correlation coefficient statistic. With the linear autoregressive process, the TTS test generally has lower power than other tests, and all tests are sensitive to erroneous choices of *l*. In S3 Data, we include all tests from Fig 2 and vary parameters such as time series length and interaction strength, again finding that the TTS test has comparatively low power. With the logistic map process, the TTS test and IAAFT test both have excellent power, although the IAAFT test is less sensitive than the TTS test to the choice of *l*. Conversely, the parametric Pearson correlation test generally has lower power, likely because the Pearson correlation coefficient is a less appropriate statistic for detecting interactions in this nonlinear system [6]. The TTS test (with cross-map skill) generally continued to have similar power as other surrogate data tests (and was superior to the parametric Pearson correlation test) when we varied parameters such as time series length and interaction strength (S4 Data). We also applied the TTS test, with mutual information as the correlation statistic, to a pair of nonlinearly coupled autoregressive processes and found lower power than other surrogate data tests but higher power than the parametric Pearson correlation test (S5 Data).

In the next 3 sections, we apply the TTS test to existing data sets from climatology, microbiome science, and animal behavior science. These case studies serve as examples of real systems wherein the TTS test is sufficiently powerful to detect dependence relationships, some (but not all) of which were detected by the original authors of the datasets.

### An example from climate science

Preindustrial climatological change is widely understood to have been driven largely by variability in the Earth’s orbit around the sun. The Earth’s rotation around the Sun is characterized by 3 parameters known as eccentricity, obliquity, and the climatic precession index (here simply “precession”). Eccentricity describes the shape of the orbit, which varies from nearly circular to slightly elliptical over a cycle with a period of approximately 96,000 years (96 kiloyears or 96 kyr). Obliquity is the angle between Earth’s rotational axis and the normal to the orbital plane, which cycles over roughly 41 kyr in a band roughly bounded between 22° and 24.5°. Precession is $esin\bar{\omega }$ where *e* is eccentricity and $\bar{\omega }$ is the longitude of perihelion (the angle between the vernal equinox and the perihelion [60]), with a cycle period of about 21 kyr [61,62]. Each of these parameters is thought to play a role in Earth’s climate, although some parameters may be more influential than others, and the extent of a parameter’s influence may change over time [62–64]. The climate record is characterized by repeated episodes of cooling followed by warming events called deglaciations. Until about 1 million years ago, deglaciations occurred with a period of about 41 kiloyears, which is the period of obliquity cycles. Because of this, obliquity is often said to “pace” glacial cycles [65,66]. Yet, 2 time series with shared periodic elements can be statistically independent (e.g., system iii of Fig 2A).

**Fig 4:**
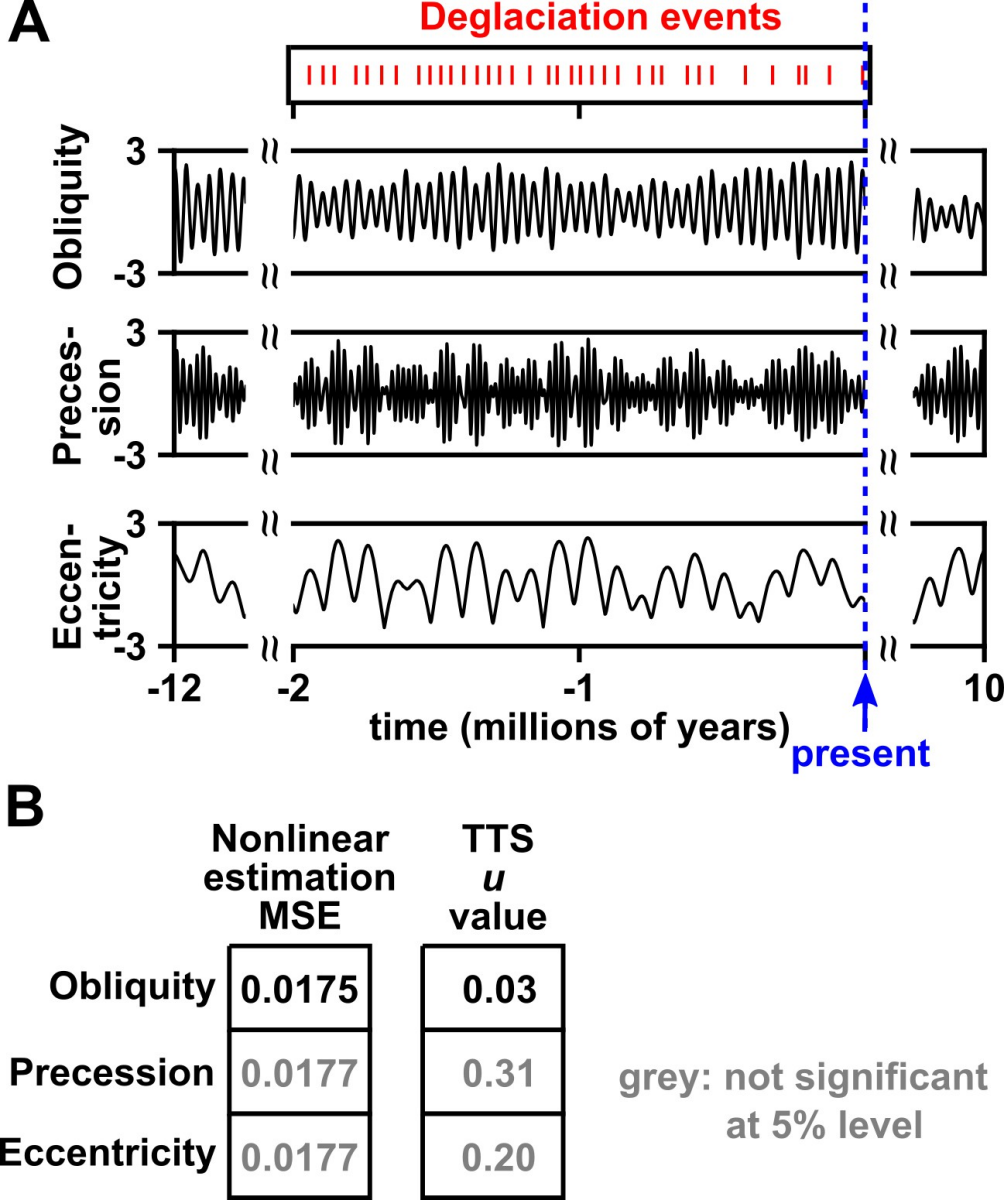
The TTS test detected dependence between deglaciation and obliquity, but not between deglaciation and precession or eccentricity. (**A**) Time series of deglaciation events (from [66]) and the 3 orbital parameters (estimated from the model in [61]). We represented the 36 deglaciation events that occurred in the last 2 million years as a time series with a sampling frequency of 1 kyr by assigning a 1 to the deglaciation variable if a kiloyear contained a deglaciation event and a 0 otherwise. Our deglaciation time series thus has 2,000 time points. We did not use a higher sampling frequency due to uncertainty in deglaciation timing and avoided a lower sampling frequency (e.g., 10 kyr) to adequately capture the shapes of the obliquity and precession cycles. To estimate obliquity, precession, and eccentricity, we used the numerical solution from [61], which provides accurate estimates of orbital parameters over at least tens of millions of years (and also predicts future values). Orbital values are standardized to a mean of 0 and variance of 1. To obtain unshifted correlations, we used truncated time series with times between –1999 kyr and 0 kyr (present time), yielding a total of 2,000 time points. Orbital parameter time series were used to generate time-shifted surrogates, with a truncation radius of 10 million years (20,000 time-shifted surrogates). (**B**) Testing for dependence between orbital parameters and deglaciation. The correlation statistic for the TTS test is the mean squared error (MSE) when using orbital parameters to estimate deglaciation events via a state space-based technique. This nonlinear statistic is similar to the cross-map skill statistic used in other largely deterministic nonlinear systems (section 8.2 of S1 Text). The numerical values shown in this figure and the code used to produce the figure are in S1 Data and S1 Code, respectively.

Using the TTS procedure, we tested for dependence between orbital parameters and deglaciations with only the assumption that the time series of the 3 orbital parameters are stationary (Fig 4A). We used the entire past 2 million years (2 Myr) of deglaciation series as our “truncated” *x* time series and generated surrogates using orbital parameter time series spanning from 12 Myr in the past to 10 Myr in the future (i.e., with a truncation radius of 10 Myr; Fig 4A). Future orbital parameter values can be used due to the availability of an accurate physical model of Earth’s orbit [61]. We use a large truncation radius because the available orbital parameter time series are far longer than the available deglaciation time series, in contrast to Fig 3 (wherein time series had equal lengths). To test for dependence, we next sought an appropriate correlation statistic. Pearson correlation seems unnatural in this context because it may be near 0 (and thus fail to detect dependence) if, for example, deglaciations occur at the midpoints between peaks and troughs of orbital series. To avoid this problem, we used a prediction-based nonlinear correlation statistic (similar but not identical to the cross-map skill statistic used in Fig 3E; see section 8.2 of S1 Text for more details). Using this statistic, the TTS test detected a dependence between deglaciation times and obliquity (*u*<0.05), but not the other 2 orbital parameters (Fig 4B), similar to Huybers’ original period-based analysis [66].

We did not incorporate lags in our test. The temporal lag between orbital parameters and temperature has been estimated in several studies [67–69]. For example, Imbrie and colleagues [67] found that during the last half-million years, ice minima tended to lag fluctuations in orbital parameters by about 9 and 6 kyr for obliquity and precession, respectively. However, since all of these estimates apply to the same system (i.e., there is only 1 Earth), using an empirically estimated lag for the TTS test would be “cheating” and could invalidate the test. On the other hand, our simulations (section 8.3 of S1 Text) suggest that the statistical power of our test might not be especially sensitive to most lags shorter than or similar length to the lags obtained by Imbrie and colleagues.

### An example from human microbiome science

The human microbiome is spatially structured, with different body sites playing host to distinct microbial communities [70]. Transfer of microbes among body sites is thought to have important consequences for human health and disease [71]. Here, we apply the TTS test to data from a year-long daily microbiome survey [72] to look for dependence relationships among the local microbiomes at different sites on the body of a healthy human individual. Such dependence relationships would be consistent with cross-site microbial transfer.

Importantly, we focus in this example on whether there is any detectable migration among body sites, rather than studying the migratory status of each individual species. This is because many microbiome surveys (including the one analyzed here [72]) measure only the relative abundance of different species, and this makes it difficult to perform species-level analyses (Fig A-17 in S1 Text; see also [73]). Put simply, the relative abundances of independent species will be dependent (since they sum to 1). However, testing for overall dependence among body sites is not impeded by the nature of relative abundance data. Intuitively, this is because if the absolute abundances of microbial species on 2 body sites are independent, then the relative abundances must also be independent. As this statement’s contrapositive, if the relative abundances of microbial species on 2 body sites are dependent, then the absolute abundances must also be dependent (see section 9.2 of S1 Text for a formal argument).

**Fig 5:**
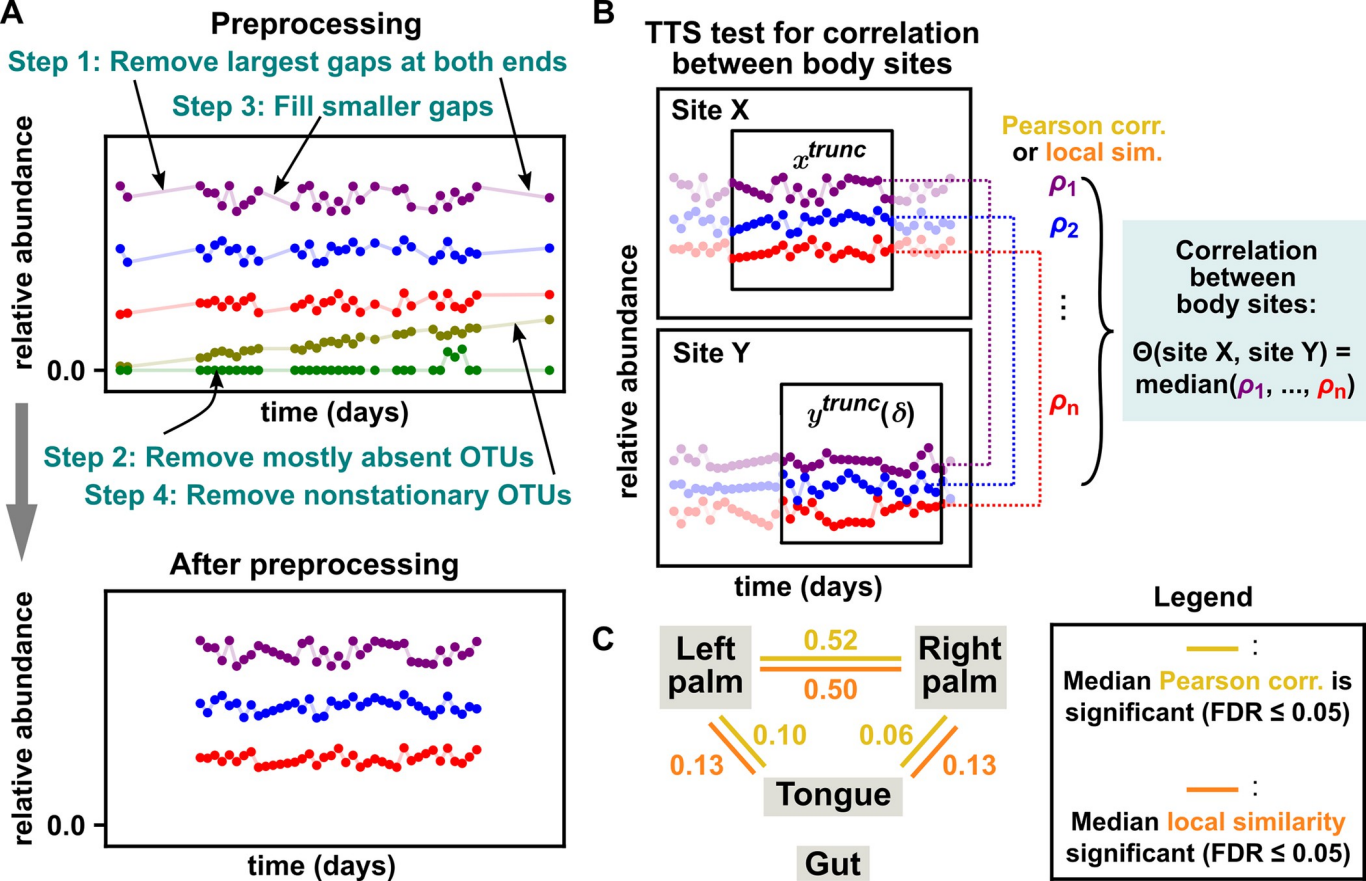
The TTS test applied to longitudinally sampled microbiomes from 4 body sites detects dependence between body sites. (**A**) Data preprocessing. To remove long (>6-day) gaps at the beginning and end of time series, we only used measurements from day 42 to day 418. Remaining gaps were filled by linear interpolation or by randomly resampling abundance values. OTUs were removed if they were absent in over half of measurements or if they were not considered stationary (at the 0.05 significance level) by an ADF test implemented in the Statsmodels Python package [75]. OTUs removed from 1 body site were not necessarily removed from the other body sites so that correlations could still be computed between the other sites. (**B**) TTS test procedure for correlation between body sites. We used an intermediate truncation radius *r* of 79 days (inspired by Fig 3C). We quantified correlation between 2 body sites as follows: For each shared OTU *i*, we computed *ρ*_*i*_ (the Pearson correlation or local similarity score of OTU *i* between the 2 sites). We then chose the median of {*ρ*_1_, …, *ρ*_*m*_} (where *m* is the total number of shared OTUs) to be the between-site correlation (i.e., our “Θ” in the notation of Fig 1). This setup avoids the need to perform a separate test for each OTU. (**C**) The TTS test detected dependence between the 2 palms and between palms and the tongue. Numbers denote the median intraspecies correlation as measured by Pearson correlation (in gold) or local similarity (in orange) when gaps were filled by linear interpolation. All links shown were detected with a significance level of 0.05 after a Benjamini–Hochberg FDR adjustment for performing 6 tests [76,77]. Note that the gut shares few species with the other sites (A-19 in S1 Text). The same network of significant correlations was obtained for C regardless of either the gap-filling method in A, whether Pearson correlation versus local similarity was used in B, or which sites were used to generate surrogates in the 6 pairwise tests. See section 9.6 of S1 Text for more details. The numerical values shown in this figure and the code used to produce the figure are in S1 Data and S1 Code, respectively. Panels A and B are for illustration and do not contain real data. ADF, augmented Dickey–Fuller; FDR, false discovery rate; OTU, operational taxonomic unit; TTS, truncated time-shift.

We applied the TTS test to the time series from [72] to look for dependence between the microbial communities living on the left palm, right palm, tongue, and gut. We first obtained OTU (operational taxonomic unit) relative abundance tables from the data in [72] using the online Qiita platform [74] (see section 9.4 of S1 Text). We then preprocessed data (Fig 5A) by removing or filling gaps in time series and by removing OTU abundance time series that were either mostly absent or likely nonstationary. Analyzing nonstationary processes is an important problem, but requires strong assumptions, and is outside the scope of this example. After preprocessing, the number of remaining OTUs ranged from 180 (tongue) to 507 (right palm). Finally, we performed a TTS test between each pair of body sites. Note that this example deviates from earlier examples in that both the {*x*_*t*_} and {*y*_*t*_} time series are now multivariate (due to the existence of multiple species; Fig 5B), but the TTS test still applies (section 2 of S1 Text).

To correlate datasets from 2 body sites (Fig 5B), we first listed all of their shared OTUs. Then, for each shared OTU, we computed the sample Pearson correlation coefficient between the relative abundance series of that OTU in the 2 sites. Our correlation statistic Θ was the median of these correlation coefficients across all shared OTUs. This statistic has the natural interpretation as the cross-site correlation for the “typical” species. Moreover, in simulations that capture the expected properties of the microbial time series (e.g., microbe transfers between sites occurring more frequently than the once-per-day sampling frequency; relative abundance data), correlations tend to be positive and thus taking a median does not cancel out positive and negative correlations (section 9.3 of S1 Text). We did not incorporate lags in our TTS test because we expect dependence among body sites to be largely driven by migration on time scales likely faster than the sampling period of 1 day.

We detected dependence between the microbial communities on the left and right palms and between the palms and the tongue (Fig 5C). This result was the same if instead of Pearson correlation, we used local similarity, a correlation statistic designed to detect transient temporal correlations that is popular in microbiome science [5,78]. These results reveal more cross-site dependences than the original analysis of [72], which detected dependence only between the left and right palms. The original study first computed the phylogenetic distance between temporally adjacent microbiomes within body sites and then calculated correlations between the phylogenetic distances of different body sites [72]. Whereas that analysis relied on parametric tests with assumed null distributions, the present analysis relies on correlation statistics (i.e., the median of many Pearson correlation coefficients or local similarity scores) whose parametric sampling distributions are, to our knowledge, unknown. Yet, due to the flexibility of the TTS test, we are nevertheless able to perform a valid statistical test, assuming only that abundance time series are stationary.

### An example from animal behavior science

A major goal of animal behavior research is to understand the rules that govern how animals act at the levels of both individuals and groups (e.g., swarms of insects or shoals of fish). Video tracking techniques enable measurements of variables such as an individual’s position and velocity [79]. These quantitative measurements have helped researchers detect subtle or complex behaviors, enable useful analogies between animal group behavior and materials physics [80,81], and connect individual-level and group-level phenomena [79,82–84].

**Fig 6:**
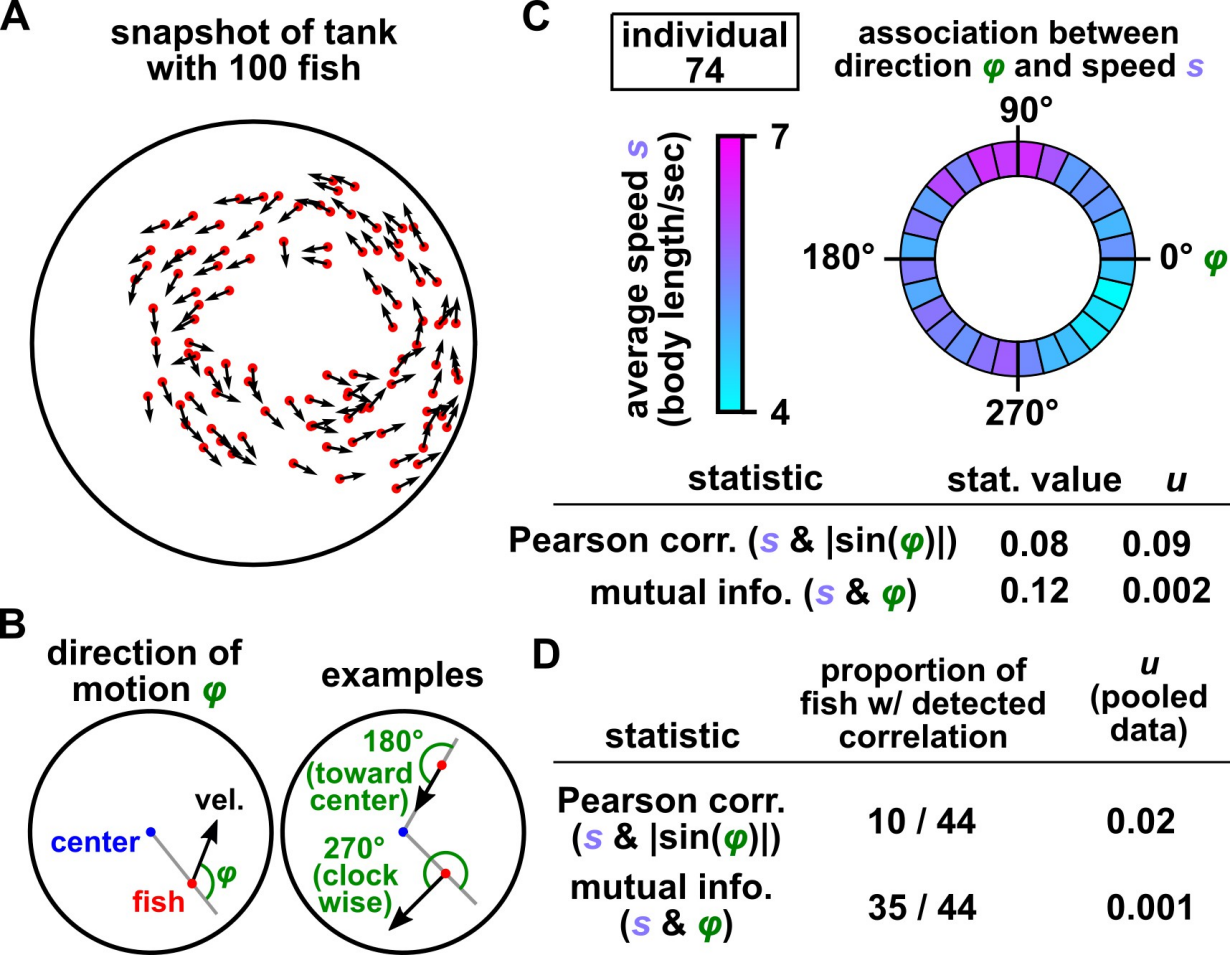
The TTS test detects dependence between swimming speed and direction in zebrafish. (**A**) A snapshot of fish positions in a 100-fish tank. (**B**) A fish’s direction of motion (*φ*) is defined as the angle between the fish’s position vector and velocity vector. Direction values of *φ* = 0° and 180° correspond to motion exactly away from the center or toward the center, respectively. Direction values of *φ* = 90° and 270° correspond to motion exactly counterclockwise and clockwise, respectively. (**C**, **D**) Association between direction (*φ*) and speed (*s*) for an arbitrary individual (“individual 74”) and for 44 fish in which recorded trajectories did not contain gaps. See A-21 in S1 Text for time series. The TTS procedure tests for dependence using the absolute value of the Pearson correlation coefficient between *s* and |sin(*φ*)|, or using the mutual information between *s* and *φ*. Mutual information detects correlations more readily in this case. When testing for correlations in 44 fish (D), detections were made at a significance level of 0.05 with a Benjamini–Hochberg FDR correction [76,77] (middle column). We also performed a TTS test that incorporated all 44 trajectories (right column; “pooled data”). This test was analogous to the test of Fig 5B; i.e., for each time shift, we calculated the overall correlation between speed and direction as the median correlation among all 44 trajectories. For all tests, we used data from the first of the 3 replicate videos in [85] and limited our analysis to the first 10^4^ frames (about 5 minutes) since this data segment appeared approximately stationary by visual inspection, although fish speeds are likely nonstationary overall (see section 10.2 of S1 Text and [86]). We used the speed variable for time-shifted surrogates and used a truncation radius of one-tenth the total time series length (1,000 frames). See section 10 of S1 Text for further details. The numerical values shown in this figure and the code used to produce the figure are in S1 Data and S1 Code, respectively.

We applied the TTS test to a set of zebrafish trajectories recorded by Romero-Ferrero and collegaues [85]. In a shallow circular tank, 100 juvenile zebrafish were placed and tracked by an overhead video camera at 32 frames per second, yielding 2D trajectories of each fish (height was not measured; Fig 6A). We here use the TTS test to ask whether there is a dependence between the speed of a fish and its direction of motion.

To define a coordinate system for direction of motion, we noticed that large groups of fish often swam parallel to the perimeter of the tank (Fig 6A). To capture this behavior, we define an individual’s direction of motion *φ* as the angle between the individual’s position vector and velocity vector (Fig 6B).

For an arbitrary fish, different directions appear to correspond to different speeds (Fig 6C). It is unnatural to quantify this association using the Pearson correlation between speed and direction *φ* because speed is a linear quantity, whereas direction is a circular quantity. We can make linear correlation more appropriate by first transforming the direction variable *φ* to |sin(*φ*)|, which is the largest when *φ* = 90° or 270° (swimming parallel to the perimeter), and the smallest when *φ* = 0° or 180° (swimming away from or toward the center), and then compute the Pearson correlation between |sin(*φ*)| and speed. Alternatively, we can use mutual information as our statistic, which is not limited to linear dependence. Fig 6C shows the statistic values and TTS test results for 1 arbitrary fish. For this fish, mutual information between speed *s* and direction *φ* is significant, while Pearson correlation between *s* and |sin(*φ*)| is not (Fig 6C). Overall, the TTS test detected dependence between speed and direction in 10 out of 44 fish using Pearson correlation and in 35/44 using mutual information (at the 0.05 level after a Benjamini–Hochberg false discovery rate correction), respectively (Fig 6D). The TTS tests with either statistic detected dependence in pooled data (Fig 6D). We did not incorporate lags in our test because we had no prior expectation of a coupling delay between swimming speed and direction.

## Discussion

A statistical hypothesis test for dependence between 2 time series requires a correlation statistic and a null model. These 2 ingredients seem to have received different levels of attention over the past 2 decades. Recent years have witnessed the development and rapid adoption of new correlation statistics that can detect transient or nonlinear forms of dependence, some of which even attempt to infer the direction of causation [5,6,12,13,59,78,87]. In practice, however, these correlation statistics have often been paired with inappropriate surrogate data null models. For example, analyzing an arbitrary non-Gaussian time series with the popular IAAFT surrogate procedure can be dangerous (Fig 2B, rows ii-viii, IAAFT columns; see also [27,88]). Even more concerning, studies have tested dependence hypotheses in time series using a naive permutation test, which assumes that a time series consists of independent and identically distributed data. Overall, the general problem of assigning statistical significance to nonlinear correlations between time series does not appear to have a broadly accepted solution [8,88,89].

The TTS can serve as a (provably) conservative solution to this problem since it is valid as long as one of the time series is stationary (or can be made stationary by detrending; see section 5 of S1 Text). This is a minimally restrictive requirement among valid nonparametric tests of dependence between time series. This test was sufficiently powerful to verify the previously observed dependence between obliquity and deglaciation timing (Fig 4) as well as dependence between the microbiome compositions of the left and right palms (Fig 5). In the microbiome dataset, we could even use it to identify additional relationships that went undetected by the original analysis of Caporaso and colleagues [72]. Importantly, the TTS can be applied in cases where linear parametric tests are particularly unnatural such as when correlating fish swimming speed (which lies on the number line) to direction (which lies on the circle between 0 and 2*π*). Since surrogate data tests are presently used in disciplines ranging from Earth System science to physiology [13,23,25,29,36–38,90,91], we expect the TTS test to find utility in diverse application domains.

Comparing the TTS test with similar surrogate data tests, one observes a tradeoff between rigor and power. On the one hand, the TTS test offers a guarantee of false positive rate control under a minimally restrictive assumption. On the other hand, other tests with more restrictive validity conditions (or without clear validity conditions) offer greater statistical power. Nevertheless, in at least some scenarios, when sufficient data are available and the range of possible coupling lags is known, a multi-lag TTS test can be used with power approaching 100% (e.g., Fig 3; S6 Data and S7 Data).

### Limitations of this study and the TTS test

Readers should be aware of some limitations of our benchmark simulations and of the TTS test. Our statistical power benchmark studies used ground-truth systems with relatively simple interaction mechanisms, most commonly with a single interaction term at a single lag. At most, *x* influenced *y* at 1 lag and *y* influenced *x* at a possibly different lag (S6 and S7 Data). We did not consider, e.g., settings where one variable influences another at 5 different lags spaced far apart in time. This would likely produce several peaks in the shifted correlation chart, which may require different strategies for detection. In particular, for a single (not multi-shift) TTS test, even if *θ*_0_ occurred on one of the peaks, *r* may need to be large enough to account for other potentially higher peaks.

Multiple correlation peaks can also occur when one or both time series exhibit certain forms of long-range dependence, such as periodic behavior. In the extreme case where *x* and *y* are phased-synchronized periodic processes with a frequency above the significance level, the TTS will never be able to detect dependence. However, in section 11.1 of S1 Text, we show that the same pathology is observed for any test that is nonrandom (meaning that the same data will always generate the same test result) and has the same null hypothesis and assumptions as the TTS test.

Similarly, the TTS test has limited power when we do not have sufficient data to handle an unknown and potentially large coupling delay, which would require the multi-lag procedure. Recall that per Eq 5, a multi-lag TTS test with *m* lags requires a truncation radius of at least *m*/*α*−1 and thus requires a time series longer than 2(*m*/*α*−1).

For ease of presentation, Fig 3 examined systems where the interaction lag in the ground-truth equations was also the lag that maximized correlation. Although this idea is intuitive, there are exceptions. For example, with certain parameter choices, bidirectionally coupled logistic maps with interaction lags of 1 can have correlations that are routinely maximized at lags of 5 or more (pg. 42 in [12]).

Since the stationarity assumption is central in dependence testing, a statistical test for stationarity would be convenient. However, a single time series can in theory be described by either a stationary process (e.g., Fig A-22A in S1 Text) or a nonstationary one (e.g., Fig A-22B in S1 Text). Nevertheless, many methods attempt to test for stationarity or a similar property in a single time series, albeit with various modeling assumptions or other caveats [92–95]. For example, although the popular augmented Dickey–Fuller (ADF) test [92,95] is sometimes used as a pragmatic means of assessing stationarity (e.g., Fig 4; [96,97]), its null hypothesis is not exactly nonstationarity. In fact, a rejection of the ADF test’s null hypothesis indicates that a time series is free from some sources of nonstationarity (e.g., a random walk), but other sources of nonstationarity (e.g., time-varying parameters) may still be present. Statistical tests cannot guarantee that a time series is stationary but can provide supporting evidence. Scientific knowledge can also be used to support the stationarity assumption: The stationarity condition is often met by stochastic processes that tend to relax toward a stable equilibrium [98]. Periodic processes, measurement noise processes, and chaotic processes can also be stationary (e.g., Fig 2).

### Future directions and outlook

The subject of conditional dependence has been conspicuously absent from our discussion. Tests of conditional dependence (i.e., whether 2 variables are dependent after we statistically control for a third variable) can help to rule out possible common-cause explanations and can sometimes even be used to reveal the direction of causation [12,99]. We initially motivated the TTS test by noticing that it enjoys the rigor and generality of the permutation test but applies to time series rather than iid data. Could a test for conditional dependence between time series be devised with the same rigor and generality? This seems difficult. Even for continuous iid data, it has been proven under general conditions that no valid test for conditional dependence can both avoid making assumptions and have statistical power [100]. Thus, we expect most tests of conditional dependence among time series to be less rigorous or require more assumptions than the TTS test. Nevertheless, there have been promising recent advances on this front, such as a test based on constrained shuffling [101,102] and the “knockoff” testing approach, which has been recently applied to sequential data [103,104]. Exploring methods that can robustly test for conditional dependence between time series is an important future direction.

In sum, an important and long-standing problem is that of nonparametrically testing for the statistical significance of a correlation between autocorrelated datasets such as time series. The TTS test provides an approach that imposes relatively minimal requirements onto both the correlation statistic and the data-generating process. This gives researchers the freedom to apply a large arsenal of correlation statistics to a wide array of processes without sacrificing test validity. This freedom will become more valuable in the future as both correlation techniques and data availability continue to proliferate across diverse fields of research.

## Methods

### Surrogate data tests for comparative benchmarks

For surrogate data tests based on the IAAFT, stationary block bootstrap, or twin procedures, we used 499 surrogates for the empirical null distribution, unless otherwise specified. Custom Python scripts were used to generate stationary block bootstrap surrogates, cyclic permutation surrogates, TTS surrogates, and naive TTS surrogates. We used the Pyunicorn package [105] to generate IAAFT and twin surrogates.

For IAAFT surrogates [28], we used the “refined_AAFT_surrogates” function in Pyunicorn. We set the “n_iterations” argument to 200 and the “output” argument to “true_spectrum.” For stationary block bootstrap surrogates, we set the parameter known as *p* in [56] to 0.05. This parameter sets the average block length, which is approximately 1/*p*. Cyclic permutation surrogates were generated by shifting in time with a wraparound. If the original time series was of length *n*, then *n-1* cyclic permutation surrogate were produced. For example, if the original time series was {*x*_1_,*x*_2_,*x*_3_,*x*_4_}, then there would be 3 cyclic permutation surrogates: {*x*_2_,*x*_3_,*x*_4_,*x*_1_}, {*x*_3_,*x*_4_,*x*_1_,*x*_2_}, and {*x*_4_,*x*_1_,*x*_2_,*x*_3_}.

For twin surrogates [33], we used the “twin_surrogates” function in the Pyunicorn package. This function requires 4 parameter arguments: the minimum temporal distance between twins, the delay vector lag, the delay vector embedding dimension, and the “recurrence threshold.” We set the minimum distance between twins to 1. To choose the delay vector lag and embedding dimension, we used the function “takens_embedding_optimal_parameters” in the Python package giotto-tda [106]. Briefly, this function first selects the delay lag *τ* so that the mutual information between values *τ* steps apart is minimized. The function then selects the embedding dimension by the widely used “false nearest neighbors” algorithm, which is difficult to describe concisely but is explained clearly by its inventors [107]. The function “takens_embedding_optimal_parameters” requires 2 arguments, the maximum delay lag and the maximum embedding dimension, and these were both set to 8. Finally, we chose the recurrence threshold parameter by a method advocated by the original authors of the twin procedure [33], which is to select the recurrence threshold that causes the recurrence plot to have between 5% and 20% “black points.” We used 12% black points as this is in the middle of the recommended range.

Following previous works [13,28], we preprocessed time series to reduce a potential mismatch between the earliest and latest times before applying certain surrogate data tests. We used this preprocessing step (henceforth called “circularization”) for 3 surrogate procedures: IAAFT, cyclic permutation, and stationary block bootstrap. Circularization is recommended for IAAFT surrogates to avoid artifacts due to the periodic nature of Fourier components, and it is recommended for cyclic permutation surrogates because they directly join the beginning and ends of the time series [13]. We also used circularization for the stationary block bootstrap because this technique also sometimes join the extremes of the time series.

To circularize a time series {*y*_1_,*y*_2_,…,*y*_*n*_}, we truncate it to $\left\{y_{k_{start}},y_{k_{start}+1},\ldots ,y_{k_{end}-1}\right\}$, where *k*_*start*_ and *k*_*end*_ are chosen to minimize the mismatch between the beginning and end of the truncated time series using a formula quoted in [13]:

$$(k_{start},k_{end})=\underset{\left(k_{1},k_{2}\right)}{argmin}\left(\sum_{i=0}^{L}(y_{k_{2}+i}-y_{k_{1}+i}{)}^{2}\right).$$ (6)

We used *L* = 10. Additionally, to ensure that the circularized time series is not too short, we require that *k*_1_ and *k*_2_ be near the beginning and end of the time series, respectively. Specifically, we impose the constraints *k*_1_≤40 and *n*−*L*−*k*_2_+1≤40.

When circularization was used, *k*_*start*_ and *k*_*end*_ were chosen based on the time series that was used to generate surrogates, but both the *x* and *y* time series were circularized using the same choice of *k*_*start*_ and *k*_*end*_. Additionally, both the original and surrogate correlations were calculated from circularized time series. Circularization generally improved the false positive rates of tests (compare Fig 2 to Fig A-9 in S1 Text).

### Parametric significance test

We used a parametric test described by [19]. Under the null hypothesis that 2 stochastic processes {*x*_1_,…,*x*_*n*_} and {*y*_1_,…,*y*_*n*_} are independent, this method estimates the variance of the sample Pearson correlation coefficient (denoted ${\hat{\sigma }}_{\rho }^{2}$) as:

$${\hat{\sigma }}_{\rho }^{2}=\frac{\sum_{k=0}^{n-1}n_{k}{\hat{C}}_{x}(k){\hat{C}}_{y}(k)}{n^{2}{\hat{\sigma }}_{x}^{2}{\hat{\sigma }}_{y}^{2}}$$

where ${\hat{C}}_{x}(k)$ is the estimated autocovariance of the time series *x* at lag *k*:

$${\hat{C}}_{x}(k)=\frac{1}{n-k}\sum_{t=1}^{n-k}(x_{t}-\bar{x})(x_{t+k}-\bar{x});\;\bar{x}=\frac{1}{n}\sum_{t=1}^{n}x_{t}$$

and ${\hat{\sigma }}_{x}^{2}$ is the estimated variance of time series *x*:

$${\hat{\sigma }}_{x}^{2}=\frac{1}{n}\sum_{t=1}^{n}(x_{t}-\bar{x}{)}^{2}$$

and *n*_*k*_ is the number of entries *A*_*ij*_ in an *n*-by-*n* matrix *A* such that |*i*−*j*| = *k*. In other words, *n*_*k*_ = *n* if *k* = 0 and *n*_*k*_ = 2(*n*−*k*) if 0<*k*<*n*.

Since ${\hat{\sigma }}_{\rho }^{2}$ is estimated from finite data, it can under some circumstances be negative, which is nonsensical. If this occurred, ${\hat{\sigma }}_{\rho }^{2}$ was simply set to 1/*n*, as is often recommended [19,20,108]. Note that ${\hat{\sigma }}_{\rho }^{2}=1/n$ corresponds to the case without autocorrelation (to see this, plug into the equation for ${\hat{\sigma }}_{\rho }^{2}$ the following: ${\hat{C}}_{x}(k)={\hat{\sigma }}_{x}^{2}$ if *k* = 0 and ${\hat{C}}_{x}(k)=0$ otherwise).

Next, the autocorrelation-corrected “effective sample size” is given by $\hat{m}=1+{\hat{\sigma }}_{\rho }^{-2}$, and a standard *t* test of the Pearson correlation is performed using $\hat{m}$ in place of *n*; i.e., the test statistic is $T=\hat{\rho }(\hat{m}-2{)}^{1/2}/(1-{\hat{\rho }}^{2}{)}^{1/2}$, where $\hat{\rho }$ is the sample Pearson correlation coefficient, and a two-tailed *p*-value is computed by comparing *T* to a Student *t* distribution with $\hat{m}-2$ degrees of freedom [19]. This test is what is referred to as the “parametric test” unless otherwise stated.

In S2 Data, we compare the false positive rate of this test to that of a modified version. In that file, “Test 1” refers to the test described above. “Test 2” refers to a variant of this test in which ${\hat{C}}_{x}(k){\hat{C}}_{y}(k)$ is set to 0 for *k*>*n*/4, as suggested by [20,21] (but otherwise, all steps are the same as in Test 1). A rationale for this is that in many time series, ${\hat{C}}_{x}(k)$ for large values of *k* are probably small, but estimating these autocovariance terms is difficult due to sampling variation [21].

### Mutual information

To estimate mutual information (except in Fig 6; see next paragraph), we used the internal function “_compute_mi_cc” in the Scikit-Learn package [109] (available at: github.com/scikit-learn/scikit-learn/blob/2beed55847ee70d363bdbfe14ee4401438fba057/sklearn/feature_selection/_mutual_info.py#L18), which implements “estimator *I*^(1)^” of [53]. The estimator requires a choice of distance metric for each of the variables being correlated, and 1 parameter (the number of neighbors). We used 3 neighbors and regular (i.e., Euclidean) distance for both variables.

For the fish behavior example in which we correlated speed with direction (Fig 6), the circular nature of the direction variable motivated a slightly different mutual information estimator. Specifically, we again used the *I*^(1)^ estimator of [53] with 3 neighbors and used Euclidean distance for speed. For direction, we used angular distance so that, e.g., the angles of 0.1*π* and 1.9*π* would have a distance of 0.2*π* rather than 1.8*π*. Mathematically, we took the distance between 2 direction angles *α* and *β* to be

arccos(cos(α−β))=arccos(cos(α)cos(β)+sin(α)sin(β)).

This restricts the range of possible angular distances to (0,*π*). We implemented the mutual information estimator using this distance metric as a custom Python script accelerated with the Numba compiler [110].

## Supporting information

S1 Data Numerical values shown in figures in the main text and appendix (S1 Text). (ZIP)

S2 Data False positive rates of tests for Pearson correlation benchmarked against the same ground-truth systems as in Fig 2. (XLSX)

S3 Data Power of various dependence tests in a unidirectionally coupled linear autoregressive process. (XLSX)

S4 Data Power of various dependence tests in a unidirectionally coupled logistic map process. (XLSX)

S5 Data Power of various dependence tests in a nonlinearly coupled autoregressive process. (XLSX)

S6 Data Power of various dependence tests in a bidirectionally coupled linear autoregressive process with random coupling lags. (XLSX)

S7 Data Power of various dependence tests in a bidirectionally coupled logistic map process with random coupling lags. (XLSX)

S8 Data False positive rates of multi-shift TTS test in same ground-truth systems as in Fig 2. (XLSX)

S1 Code All code used to generate figures in the main text and S1 Text, and supporting data files, as well as execution instructions. (ZIP)

S1 Text Appendix. The appendix includes a formal proof of the validity of the TTS test, extended discussions of methods, and auxiliary results. It contains Figures A-1 through A-23. See the first page for a table of contents.
(PDF)

## Abbreviations

ADF: augmented Dickey–Fuller
IAAFT: iterative amplitude-adjusted Fourier transform
OTU: operational taxonomic unit
TTS: truncated time-shift

## References

[1] Yule GU. Why do we sometimes get nonsense-correlations between Time-Series?—A study in sampling and the nature of time-series. J R Stat Soc. 1926;89(1):1–63.

[2] Granger C, Newbold P. Spurious regressions in econometrics. J Econom. 1974;2(2):111–120.

[3] Weiss S, Van Treuren W, Lozupone C, Faust K, Friedman J, Deng Y, et al. Correlation detection strategies in microbial data sets vary widely in sensitivity and precision. ISME J. 2016;10(7):1669–1681. doi: 10.1038/ismej.2015.235 26905627 PMC4918442

[4] Coenen AR, Weitz JS. Limitations of Correlation-Based Inference in Complex Virus-Microbe Communities. mSystems. 2018;3(4):e00084–18. doi: 10.1128/mSystems.00084-18 30175237 PMC6113591

[5] Ruan Q, Dutta D, Schwalbach MS, Steele JA, Fuhrman JA, Sun F. Local similarity analysis reveals unique associations among marine bacterioplankton species and environmental factors. Bioinformatics. 2006;22(20):2532–2538. doi: 10.1093/bioinformatics/btl417 16882654

[6] Sugihara G, May R, Ye H, Hsieh CH, Deyle E, Fogarty M, et al. Detecting causality in complex ecosystems. Science. 2012;338(6106):496–500. doi: 10.1126/science.1227079 22997134

[7] Peters J, Janzing D, Schölkopf B. Elements of causal inference: foundations and learning algorithms. MIT press; 2017.

[8] Runge J, Bathiany S, Bollt E, Camps-Valls G, Coumou D, Deyle E, et al. Inferring causation from time series in Earth system sciences. Nat Commun. 2019;10(1):1–13. 31201306 10.1038/s41467-019-10105-3 PMC6572812

[9] Davison AC, Hinkley DV. Bootstrap Methods and their Application. Cambridge Series in Statistical and Probabilistic Mathematics. Cambridge University Press; 1997.

[10] Lehmann EL, Romano JP. Testing statistical hypotheses. Springer Science & Business Media; 2006.

[11] Conover W. Distribution-free methods in statistics. Wiley Interdiscip Rev Comput Stat. 2009;1(2):199–207.

[12] Yuan AE, Shou W. Data-driven causal analysis of observational biological time series. Elife. 2022;11:e72518. doi: 10.7554/eLife.72518 35983746 PMC9391047

[13] Lancaster G, Iatsenko D, Pidde A, Ticcinelli V, Stefanovska A. Surrogate data for hypothesis testing of physical systems. Phys Rep. 2018;748:1–60.

[14] Weinstein SM, Vandekar SN, Adebimpe A, Tapera TM, Robert-Fitzgerald T, Gur RC, et al. A simple permutation-based test of intermodal correspondence. Hum Brain Mapp. 2021;42(16):5175–5187. doi: 10.1002/hbm.25577 34519385 PMC8519855

[15] Molenaar PC. A manifesto on psychology as idiographic science: Bringing the person back into scientific psychology, this time forever. Measurement. 2004;2(4):201–218.

[16] Warner RM. Spectral analysis of time-series data. Guilford Press; 1998.

[17] Cliff OM, Novelli L, Fulcher BD, Shine JM, Lizier JT. Assessing the significance of directed and multivariate measures of linear dependence between time series. Phys Rev Research. 2021;3:013145. doi: 10.1103/PhysRevResearch.3.013145

[18] Harris KD. A Shift Test for Independence in Generic Time Series; 2020. Available from: https://arxiv.org/abs/2012.06862.

[19] Clifford P, Richardson S, Hemon D. Assessing the Significance of the Correlation between Two Spatial Processes. Biometrics. 1989;45(1):123–134. 2720048

[20] Pyper BJ, Peterman RM. Comparison of methods to account for autocorrelation in correlation analyses of fish data. Can J Fish Aquat Sci. 1998;55(9):2127–2140.

[21] Afyouni S, Smith SM, Nichols TE. Effective degrees of freedom of the Pearson’s correlation coefficient under autocorrelation. Neuroimage. 2019;199:609–625. doi: 10.1016/j.neuroimage.2019.05.011 31158478 PMC6693558

[22] Brookshire E, Weaver T. Long-term decline in grassland productivity driven by increasing dryness. Nat Commun. 2015;6(1):1–7. doi: 10.1038/ncomms8148 25972300 PMC4479003

[23] Tsonis AA, Deyle ER, May RM, Sugihara G, Swanson K, Verbeten JD, et al. Dynamical evidence for causality between galactic cosmic rays and interannual variation in global temperature. Proc Natl Acad Sci. 2015;112(11):3253–3256. doi: 10.1073/pnas.1420291112 25733877 PMC4371914

[24] Van Nes EH, Scheffer M, Brovkin V, Lenton TM, Ye H, Deyle E, et al. Causal feedbacks in climate change. Nat Clim Change. 2015;5(5):445–448.

[25] Matsuzaki SiS, Suzuki K, Kadoya T, Nakagawa M, Takamura N. Bottom-up linkages between primary production, zooplankton, and fish in a shallow, hypereutrophic lake. Ecology. 2018;99(9):2025–2036. doi: 10.1002/ecy.2414 29884987

[26] Wang M, Yoshimura C, Allam A, Kimura F, Honma T. Causality analysis and prediction of 2-methylisoborneol production in a reservoir using empirical dynamic modeling. Water Res. 2019;163:114864. doi: 10.1016/j.watres.2019.114864 31330398

[27] Andrzejak RG, Kraskov A, Stögbauer H, Mormann F, Kreuz T. Bivariate surrogate techniques: necessity, strengths, and caveats. Phys Rev E. 2003;68(6):066202. doi: 10.1103/PhysRevE.68.066202 14754292

[28] Schreiber T, Schmitz A. Surrogate time series. Phys D Nonlinear Phenom. 2000;142(3–4):346–382.

[29] Ebisuzaki W. A method to estimate the statistical significance of a correlation when the data are serially correlated. J Climate. 1997;10(9):2147–2153.

[30] Chan KS. On the validity of the method of surrogate data. Fields Inst Commun. 1997;11:77–97.

[31] Diks C, DeGoede J. A general nonparametric bootstrap test for Granger causality. Global analysis of dynamical systems; 2001, p. 391–403.

[32] Papana A, Kyrtsou C, Kugiumtzis D, Diks C. Assessment of resampling methods for causality testing: A note on the US inflation behavior. PLoS ONE. 2017;12(7):e0180852. doi: 10.1371/journal.pone.0180852 28708870 PMC5510825

[33] Thiel M, Romano MC, Kurths J, Rolfs M, Kliegl R. Twin surrogates to test for complex synchronisation. Europhys Lett. 2006;75(4):535.

[34] Romano MC, Thiel M, Kurths J, Mergenthaler K, Engbert R. Hypothesis test for synchronization: twin surrogates revisited. Chaos. 2009;19(1):015108. doi: 10.1063/1.3072784 19335012

[35] Jia Z, Lin Y, Liu Y, Jiao Z, Wang J. Refined nonuniform embedding for coupling detection in multivariate time series. Phys Rev E. 2020;101(6):062113. doi: 10.1103/PhysRevE.101.062113 32688603

[36] Netoff TI, Schiff SJ. Decreased neuronal synchronization during experimental seizures. J Neurosci. 2002;22(16):7297–7307. doi: 10.1523/JNEUROSCI.22-16-07297.2002 12177225 PMC6757884

[37] Quiroga RQ, Kraskov A, Kreuz T, Grassberger P. Performance of different synchronization measures in real data: a case study on electroencephalographic signals. Phys Rev E. 2002;65(4):041903. 10.1103/PhysRevE.65.041903 12005869

[38] Faes L, Porta A, Nollo G. Mutual nonlinear prediction as a tool to evaluate coupling strength and directionality in bivariate time series: comparison among different strategies based on k nearest neighbors. Phys Rev E. 2008;78(2):026201. doi: 10.1103/PhysRevE.78.026201 18850915

[39] Vlachos I, Kugiumtzis D. Nonuniform state-space reconstruction and coupling detection. Phys Rev E. 2010;82(1):016207. doi: 10.1103/PhysRevE.82.016207 20866707

[40] Bartlett M. Some aspects of the time-correlation problem in regard to tests of significance. J R Stat Soc. 1935;98(3):536–543.

[41] Yuan AE, Shou W. An exactly valid and distribution-free statistical significance test for correlations between time series. bioRxiv [Preprint]. 2022. doi: 10.1101/2022.01.25.477698

[42] Stout WF. Almost Sure Convergence. Probability and Mathematical Statistics. Academic Press; 1974.

[43] Lindgren G. Stationary stochastic processes: theory and applications. CRC Press; 2012.

[44] Greene WH. Econometric Analysis. Pearson; 2012.

[45] Modica G, Poggiolini L. A first course in probability and Markov Chains. John Wiley & Sons; 2012.

[46] Ramdas AK, Barber RF, Wainwright MJ, Jordan MI. A unified treatment of multiple testing with prior knowledge using the p-filter. Ann Stat. 2019;47(5):2790–2821.

[47] Chen X, Doerge RW, Sarkar SK. A weighted FDR procedure under discrete and heterogeneous null distributions. Biom J. 2020;62(6):1544–1563. doi: 10.1002/bimj.201900216 32367597

[48] FitzHugh R. Impulses and physiological states in theoretical models of nerve membrane. Biophys J. 1961;1(6):445–466. doi: 10.1016/s0006-3495(61)86902-6 19431309 PMC1366333

[49] Vano J, Wildenberg J, Anderson M, Noel J, Sprott J. Chaos in low-dimensional Lotka–Volterra models of competition. Nonlinearity. 2006;19(10):2391.

[50] Tjøstheim D. Non-linear time series and Markov chains. Adv Appl Probab. 1990;22(3):587–611.

[51] May RM. Simple mathematical models with very complicated dynamics. The Theory of Chaotic Attractors; 2004, p. 85–93.

[52] Weisstein EW. Sawtooth Wave. From MathWorld—A Wolfram Web Resource. Available from: https://mathworld.wolfram.com/SawtoothWave.html.

[53] Kraskov A, Stögbauer H, Grassberger P. Estimating mutual information. Phys Rev E. 2004;69(6):066138. 10.1103/PhysRevE.69.066138 15244698

[54] Granger CW. Testing for causality: a personal viewpoint. J Econ Dyn Control. 1980;2:329–352.

[55] Shorten DP, Spinney RE, Lizier JT. Estimating transfer entropy in continuous time between neural spike trains or other event-based data. PLoS Comput Biol. 2021;17(4):e1008054. doi: 10.1371/journal.pcbi.1008054 33872296 PMC8084348

[56] Politis DN, Romano JP. The stationary bootstrap. J Am Stat Assoc. 1994;89(428):1303–1313.

[57] Lucio J, Valdés R, Rodríguez L. Improvements to surrogate data methods for nonstationary time series. Phys Rev E. 2012;85(5):056202. doi: 10.1103/PhysRevE.85.056202 23004838

[58] Davidson R, MacKinnon JG. Bootstrap tests: How many bootstraps? Econom Rev. 2000;19(1):55–68.

[59] Ye H, Deyle ER, Gilarranz LJ, Sugihara G. Distinguishing time-delayed causal interactions using convergent cross mapping. Sci Rep. 2015;5:14750. doi: 10.1038/srep14750 26435402 PMC4592974

[60] Paillard D. Glacial cycles: toward a new paradigm. Rev Geophys. 2001;39(3):325–346.

[61] Laskar J, Robutel P, Joutel F, Gastineau M, Correia A, Levrard B. A long-term numerical solution for the insolation quantities of the Earth. Astron Astrophys. 2004;428(1):261–285.

[62] Maslin M. Forty years of linking orbits to ice ages. Nature 2016;540(7632):208–209. 27929020 10.1038/540208a

[63] Hays JD, Imbrie J, Shackleton NJ. Variations in the Earth’s orbit: pacemaker of the ice ages. Science. 1976;194(4270):1121–1132. doi: 10.1126/science.194.4270.1121 17790893

[64] Lorius C, Jouzel J, Ritz C, Merlivat L, Barkov N, Korotkevich YS, et al. A 150,000-year climatic record from Antarctic ice. Nature. 1985;316(6029):591–596.

[65] Huybers P, Wunsch C. Obliquity pacing of the late Pleistocene glacial terminations. Nature. 2005;434(7032):491–494. doi: 10.1038/nature03401 15791252

[66] Huybers P. Glacial variability over the last two million years: an extended depth-derived agemodel, continuous obliquity pacing, and the Pleistocene progression. Quat Sci Rev. 2007;26(1–2):37–55.

[67] Imbrie J, Boyle E, Clemens S, Duffy A, Howard W, Kukla G, et al. On the structure and origin of major glaciation cycles 1. Linear responses to Milankovitch forcing. Paleoceanography. 1992;7(6):701–738.

[68] Tuenter E, Weber S, Hilgen F, Lourens L, Ganopolski A. Simulation of climate phase lags in response to precession and obliquity forcing and the role of vegetation. Clim Dyn. 2005;24:279–295.

[69] Lisiecki LE, Raymo ME. A Pliocene-Pleistocene stack of 57 globally distributed benthic *δ*18O records. Paleoceanography. 2005;20(1):PA1003.

[70] Huttenhower C, Gevers D, Knight R, Abubucker S, Badger JH, Chinwalla AT, et al. Structure, function and diversity of the healthy human microbiome. Nature. 2012;486(7402):207. doi: 10.1038/nature11234 22699609 PMC3564958

[71] Khor B, Snow M, Herrman E, Ray N, Mansukhani K, Patel KA, et al. Interconnections between the oral and gut microbiomes: reversal of microbial dysbiosis and the balance between systemic health and disease. Microorganisms. 2021;9(3):496. doi: 10.3390/microorganisms9030496 33652903 PMC7996936

[72] Caporaso JG, Lauber CL, Costello EK, Berg-Lyons D, Gonzalez A, Stombaugh J, et al. Moving pictures of the human microbiome. Genome Biol. 2011;12(5):1–8. doi: 10.1186/gb-2011-12-5-r50 21624126 PMC3271711

[73] Gloor GB, Macklaim JM, Pawlowsky-Glahn V, Egozcue JJ. Microbiome datasets are compositional: and this is not optional. Front Microbiol. 2017;8:2224. doi: 10.3389/fmicb.2017.02224 29187837 PMC5695134

[74] Gonzalez A, Navas-Molina JA, Kosciolek T, McDonald D, Vázquez-Baeza Y, Ackermann G, et al. Qiita: rapid, web-enabled microbiome meta-analysis. Nat Methods. 2018;15(10):796–798. doi: 10.1038/s41592-018-0141-9 30275573 PMC6235622

[75] Seabold S, Perktold J. statsmodels: Econometric and statistical modeling with python. 9th Python in Science Conference; 2010.

[76] Benjamini Y, Hochberg Y. Controlling the false discovery rate: a practical and powerful approach to multiple testing. J R Stat Soc B Methodol. 1995;57(1):289–300.

[77] Benjamini Y, Yekutieli D. The control of the false discovery rate in multiple testing under dependency. Ann Stat. 2001;29(4)1165–1188.

[78] Xia LC, Steele JA, Cram JA, Cardon ZG, Simmons SL, Vallino JJ, et al. Extended local similarity analysis (eLSA) of microbial community and other time series data with replicates. BMC Syst Biol. 2011;5(2):S15. doi: 10.1186/1752-0509-5-S2-S15 22784572 PMC3287481

[79] Orger MB, de Polavieja GG. Zebrafish behavior: opportunities and challenges. Annu Rev Neurosci. 2017;40:125–147. doi: 10.1146/annurev-neuro-071714-033857 28375767

[80] Attanasi A, Cavagna A, Del Castello L, Giardina I, Melillo S, Parisi L, et al. Collective behaviour without collective order in wild swarms of midges. PLoS Comput Biol. 2014;10(7):e1003697. doi: 10.1371/journal.pcbi.1003697 25057853 PMC4109845

[81] van der Vaart K, Sinhuber M, Reynolds AM, Ouellette NT. Mechanical spectroscopy of insect swarms. Sci Adv. 2019;5(7):eaaw9305. doi: 10.1126/sciadv.aaw9305 31501772 PMC6719412

[82] Reynolds A. Langevin dynamics encapsulate the microscopic and emergent macroscopic properties of midge swarms. J R Soc Interface. 2018;15(138):20170806. doi: 10.1098/rsif.2017.0806 29298958 PMC5805982

[83] Zienkiewicz AK, Ladu F, Barton DA, Porfiri M, Di Bernardo M. Data-driven modelling of social forces and collective behaviour in zebrafish. J Theor Biol. 2018;443:39–51. doi: 10.1016/j.jtbi.2018.01.011 29366823

[84] Heras FJ, Romero-Ferrero F, Hinz RC, de Polavieja GG. Deep attention networks reveal the rules of collective motion in zebrafish. PLoS Comput Biol. 2019;15(9):e1007354. doi: 10.1371/journal.pcbi.1007354 31518357 PMC6760814

[85] Romero-Ferrero F, Bergomi MG, Hinz RC, Heras FJ, de Polavieja GG. Idtracker.ai: Tracking all individuals in small or large collectives of unmarked animals. Nat Methods. 2019;16(2):179–182. doi: 10.1038/s41592-018-0295-5 30643215

[86] Miller N, Gerlai R. From schooling to shoaling: patterns of collective motion in zebrafish (Danio rerio). PLoS ONE. 2012;7(11):e48865. doi: 10.1371/journal.pone.0048865 23166599 PMC3498229

[87] Harnack D, Laminski E, Schünemann M, Pawelzik KR. Topological causality in dynamical systems. Phys Rev Lett. 2017;119(9):098301. doi: 10.1103/PhysRevLett.119.098301 28949567

[88] Cobey S, Baskerville EB. Limits to causal inference with state-space reconstruction for infectious disease. PLoS ONE. 2016;11(12):e0169050. doi: 10.1371/journal.pone.0169050 28030639 PMC5193453

[89] Chang CW, Ushio M, Hsieh Ch. Empirical dynamic modeling for beginners. Ecol Res. 2017;32(6):785–796.

[90] Hannisdal B, Haaga KA, Reitan T, Diego D, Liow LH. Common species link global ecosystems to climate change: dynamical evidence in the planktonic fossil record. Proc R Soc B Biol Sci. 2017;284(1858):20170722. doi: 10.1098/rspb.2017.0722 28701561 PMC5524498

[91] Ushio M, Hsieh Ch, Masuda R, Deyle ER, Ye H, Chang CW, et al. Fluctuating interaction network and time-varying stability of a natural fish community. Nature. 2018;554(7692):360–363. doi: 10.1038/nature25504 29414940

[92] Dickey DA, Fuller WA. Distribution of the estimators for autoregressive time series with a unit root. J Am Stat Assoc. 1979;74(366a):427–431.

[93] Witt A, Kurths J, Pikovsky A. Testing stationarity in time series. Phys Rev E. 1998;58(2):1800.

[94] Kwiatkowski D, Phillips PC, Schmidt P, Shin Y. Testing the null hypothesis of stationarity against the alternative of a unit root: How sure are we that economic time series have a unit root? J Econom. 1992;54(1–3):159–178.

[95] Davidson R, MacKinnon JG, et al. Econometric theory and methods. Oxford University Press New York; 2004, vol. 5.

[96] Gibbons SM, Kearney SM, Smillie CS, Alm EJ. Two dynamic regimes in the human gut microbiome. PLoS Comput Biol. 2017;13(2):e1005364. doi: 10.1371/journal.pcbi.1005364 28222117 PMC5340412

[97] Odogwu NM, Onebunne CA, Chen J, Ayeni FA, Walther-Antonio MR, Olayemi OO, et al. Lactobacillus crispatus thrives in pregnancy hormonal milieu in a Nigerian patient cohort. Sci Rep. 2021;11(1):1–19. 34518588 10.1038/s41598-021-96339-y PMC8437942

[98] Jones DA, Cox DR. Nonlinear autoregressive processes. Proc R Soc Lond A Math Phys Sci. 1978;360(1700):71–95.

[99] Glymour C, Zhang K, Spirtes P. Review of causal discovery methods based on graphical models. Front Genet. 2019;10:524. doi: 10.3389/fgene.2019.00524 31214249 PMC6558187

[100] Shah RD, Peters J. The hardness of conditional independence testing and the generalised covariance measure. Ann Stat. 2020;48(3):1514–1538.

[101] Runge J. Conditional independence testing based on a nearest-neighbor estimator of conditional mutual information. International Conference on Artificial Intelligence and Statistics. PMLR; 2018. p. 938–947.

[102] Runge J, Nowack P, Kretschmer M, Flaxman S, Sejdinovic D. Detecting and quantifying causal associations in large nonlinear time series datasets. Sci Adv. 2019;5(11):eaau4996. doi: 10.1126/sciadv.aau4996 31807692 PMC6881151

[103] Candes E, Fan Y, Janson L, Lv J. Panning for gold: ‘model-X’ knockoffs for high dimensional controlled variable selection. J R Stat Soc Ser B Stat Methodol. 2018;80(3):551–577.

[104] Sesia M, Sabatti C, Candès EJ. Gene hunting with hidden Markov model knockoffs. Biometrika. 2019;106(1):1–18. doi: 10.1093/biomet/asy033 30799875 PMC6373422

[105] Donges JF, Heitzig J, Beronov B, Wiedermann M, Runge J, Feng QY, et al. Unified functional network and nonlinear time series analysis for complex systems science: The pyunicorn package. Chaos. 2015;25(11):113101. doi: 10.1063/1.4934554 26627561

[106] Tauzin G, Lupo U, Tunstall L, Pérez JB, Caorsi M, Medina-Mardones A, et al. giotto-tda: A Topological Data Analysis Toolkit for Machine Learning and Data Exploration. J Mach Learn Res. 2021;22(39):1−6.

[107] Kennel MB, Brown R, Abarbanel HD. Determining embedding dimension for phase-space reconstruction using a geometrical construction. Phys Rev A. 1992;45(6):3403. doi: 10.1103/physreva.45.3403 9907388

[108] Kope RG, Botsford LW. Determination of Factors Affecting Recruitment of Chinook Salmon Oncorhynchus tshawytscha in Central California. Fish Bull. 1990;88(2):257–269.

[109] Pedregosa F, Varoquaux G, Gramfort A, Michel V, Thirion B, Grisel O, et al. Scikit-learn: Machine Learning in Python. J Mach Learn Res. 2011;12:2825–2830.

[110] Lam SK, Pitrou A, Seibert S. Numba: A llvm-based python jit compiler. Proceedings of the Second Workshop on the LLVM Compiler Infrastructure in HPC; 2015, p. 1–6.

